# Pervasive Protein Thermal Stability Variation during the Cell Cycle

**DOI:** 10.1016/j.cell.2018.03.053

**Published:** 2018-05-31

**Authors:** Isabelle Becher, Amparo Andrés-Pons, Natalie Romanov, Frank Stein, Maike Schramm, Florence Baudin, Dominic Helm, Nils Kurzawa, André Mateus, Marie-Therese Mackmull, Athanasios Typas, Christoph W. Müller, Peer Bork, Martin Beck, Mikhail M. Savitski

**Affiliations:** 1 Genome Biology Unit, European Molecular Biology Laboratory, 69117 Heidelberg, Germany; 2 Structural and Computational Biology Unit, European Molecular Biology Laboratory, 69117 Heidelberg, Germany; 3 Proteomics Core Facility, European Molecular Biology Laboratory, 69117 Heidelberg, Germany; 4 Max Delbrück Center for Molecular Medicine, 13125 Berlin, Germany; 5 Molecular Medicine Partnership Unit, 69120 Heidelberg, Germany; 6 Department of Bioinformatics, Biocenter, University of Würzburg, 97074 Würzburg, Germany; 7 Cell Biology and Biophysics Unit, European Molecular Biology Laboratory 69117 Heidelberg, Germany

**Keywords:** thermal proteome profiling, cell cycle, proteomics

## Abstract

Quantitative mass spectrometry has established proteome-wide regulation of protein abundance and post-translational modifications in various biological processes. Here, we used quantitative mass spectrometry to systematically analyze the thermal stability and solubility of proteins on a proteome-wide scale during the eukaryotic cell cycle. We demonstrate pervasive variation of these biophysical parameters with most changes occurring in mitosis and G1. Various cellular pathways and components vary in thermal stability, such as cell-cycle factors, polymerases, and chromatin remodelers. We demonstrate that protein thermal stability serves as a proxy for enzyme activity, DNA binding, and complex formation *in situ*. Strikingly, a large cohort of intrinsically disordered and mitotically phosphorylated proteins is stabilized and solubilized in mitosis, suggesting a fundamental remodeling of the biophysical environment of the mitotic cell. Our data represent a rich resource for cell, structural, and systems biologists interested in proteome regulation during biological transitions.

## Highlights

• Proteome-wide variation of *in situ* protein thermal stability and solubility
• Thermal stability of RNA Pol II varies across the cell cycle and is DNA dependent
• Thermal profiling across the cell cycle delineates protein subcomplexes
• Intrinsically disordered proteins are less prone to aggregation during mitosis

## Graphical Abstract

**Figure:**
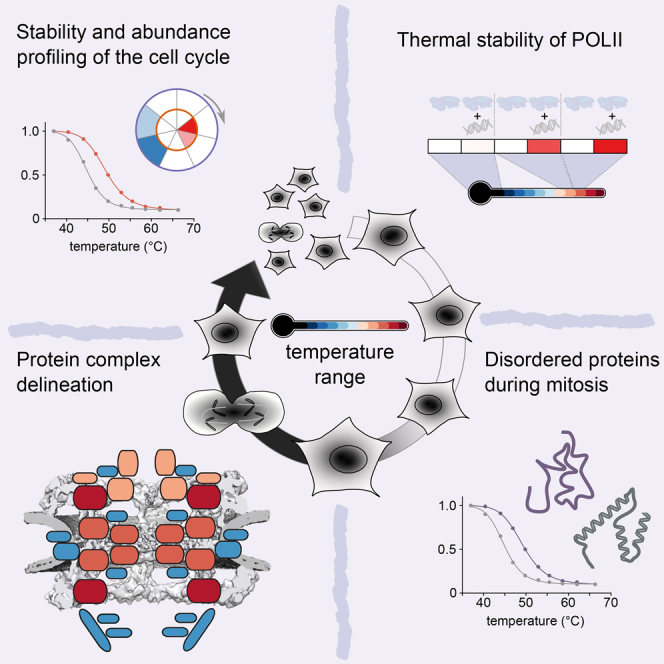

## Teaser

A proteome-wide assessment of thermal stability and solubility during the eukaryotic cell cycle demonstrates pervasive variation in mitosis and G1.

## Introduction

Mass spectrometry (MS)-based proteomics is a powerful method to accurately quantify distinct features of proteomes (Mann et al., 2013) and has provided comprehensive insight into the variability of protein abundance under distinct biological conditions, including different cell types (Geiger et al., 2012), protein complexes (Havugimana et al., 2012), and subcellular fractions (Boisvert et al., 2010). Recent advances in MS multiplexing technologies (Werner et al., 2014) enable the global measurement of protein melting curves (thermal proteome profiling; TPP) (Savitski et al., 2014). TPP and a partial digestion-based methodology for assessing protein thermal stability (Leuenberger et al., 2017) add another dimension to MS-based proteomics to quantify various states of the proteome. TPP can detect individual proteins that are stabilized *in situ* by binding to a ligand and, therefore, is useful for profiling drug targets and off-targets (Becher et al., 2016, Savitski et al., 2014). However, its utility to uncover global changes in protein thermal stability in distinct biological contexts is unclear.

The eukaryotic cell cycle is the key regulatory circuit that controls the temporal separation of fundamental processes that facilitate cell proliferation. It is well established that various aspects of proteome organization, including protein abundance and post-translational modifications, vary during cell-cycle progression (Dephoure et al., 2008, Olsen et al., 2010). We hypothesized that cell-cycle-dependent post-translational modifications, protein-protein interactions, and spatial rearrangements to distinct biophysical environments globally influence protein thermal stability (Jensen et al., 2006, Jongsma et al., 2015, Olsen et al., 2010, Pelisch et al., 2014). Here, we systematically measured *in situ* protein thermal stability, abundance, and solubility during cell-cycle progression on a proteome-wide scale.

We report the pervasive variation of protein thermal stability during the cell cycle and link it to various biological processes including transcription, spindle formation and key metabolic pathways. Further, intrinsically disordered proteins are stabilized during mitosis, coinciding with fundamental rearrangements of the proteome and the spatial outline of the cell. These changes coincide with extensive sumoylation and mitotic phosphorylation, suggesting that post-translational modifications might promote thermal stability and, in turn, prevent protein aggregation during mitotic spindle formation and chromosomal separation. Protein stabilization serves as a proxy for biological activity and complex formation, thereby revealing new players in the cell cycle. Our comprehensive analysis of cell-cycle-dependent variation of protein thermal stability, abundance, and solubility provides a valuable resource to advance the fields of transcription, structural biology, intrinsically disordered proteins, metabolism, and the cell cycle.

## Results

### Profiling the Thermal Stability, Abundance, and Solubility of Proteins during the Cell Cycle

**Figure 1:**
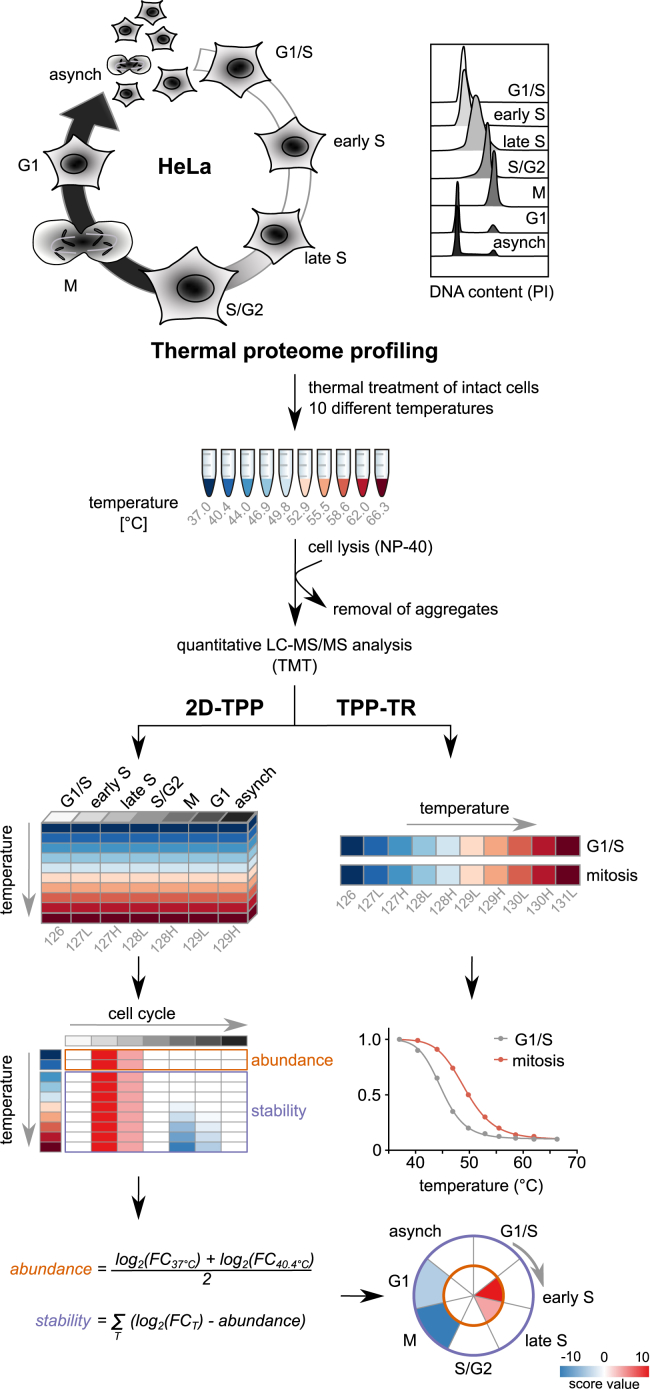
Profiling of Proteome Abundance and Stability Changes across the Cell Cycle HeLa cells were arrested with thymidine and/or nocodazole at six different cell-cycle stages, and additionally asynchronous cells were collected. For TPP experiments, intact cells were heated to ten different temperatures and lysed with NP-40. After removing protein aggregates, the soluble fractions were labeled with isobaric mass tags (TMT10-plex) for protein quantification. Samples were combined using the TPP-TR layout (right panel) for melting curve determination or in a 2D-TPP layout (left panel), in order to pool cell-cycle stages from one thermal treatment, for sensitive comparison of protein abundance and stability changes throughout the cell cycle. G1/S was used as reference point for calculating fold changes (FC). By condensing this matrix to two measures (abundance and stability scores), we identified significant changes across the proteome and represented them as a circle plot for individual proteins. Inner circle (orange), abundance changes; outer circle (purple), stability changes. See also Figure S1 and Table S1.

**Figure S1:**
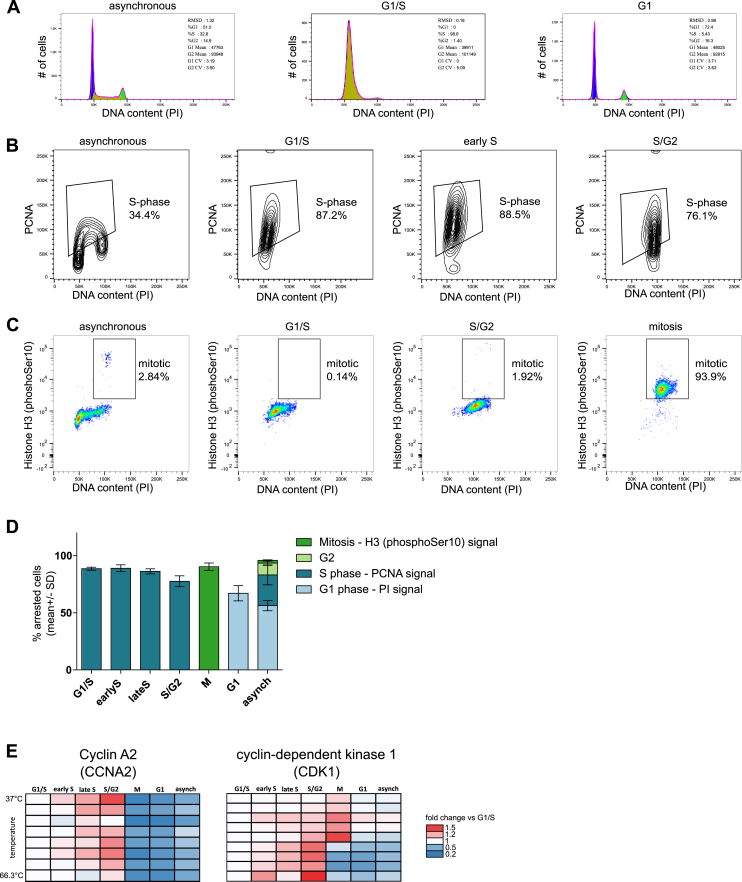
Analysis of the Different Cell-Cycle Stages in Each Sample by Flow Cytometry, Related to Figures 1 and 2A Representative images of the analysis used to quantify the amount of cells in G1-phase, S and mitosis. For all the analysis, the population of single cells with a 2n and 4n DNA content was gated based on the PI staining (pulse area versus pulse width). The G0/G1 population is shown at 50K pulse area of PI signal; the G2/M population, at 100K. (A) Histograms of DNA content. The pulse area of the PI signal was fitted to a cell cycle distribution using the Watson pragmatic model approach in FlowJo v10. The fitted populations in G1, S and G2/M stages are shown in blue, yellow and green, respectively. (B) The population with a stronger PCNA signal was gated on asynchronous cells and considered as S-phase population. The same gate was applied to all other samples. (C) To quantify the mitotic population, cells positive for phospho-Histone H3 (Ser10) and with a G2/M DNA content were gated in the asynchronous sample; this gate was subsequently applied to the rest of samples. (D) Overview of the quantification of the three replicates used for the thermal profiling experiments (2D-TPP and TPP-TR) as well as SDS extracts. The mean ± SD is shown. (E) Heatmap for CCNA2 and CDK1, from which abundance and stability scores were calculated. The vertical direction indicates the increase in temperature and the horizontal direction progress in cell cycle.

To investigate proteome variation across different stages of the cell cycle *in situ*, we used thermal proteome profiling (TPP) to measure protein thermal stability and abundance (Savitski et al., 2014). We synchronized and collected HeLa cells in six different cell-cycle stages: G1/S transition (no release after double thymidine block), early S phase (2 hr after release), late S phase (4 hr after release), S/G2 transition (6 hr after release), mitosis (0.5 hr after release from nocodazole block), and early G1 phase (4.5 hr after release from nocodazole block). We also collected asynchronous cells (Figures 1 and S1A–S1D; STAR Methods). Intact cells were heated and lysed with a mild detergent (NP-40), and soluble proteins were identified by multiplexed quantitative MS based on tandem mass tag (TMT) labeling (Werner et al., 2014).

**Figure 2:**
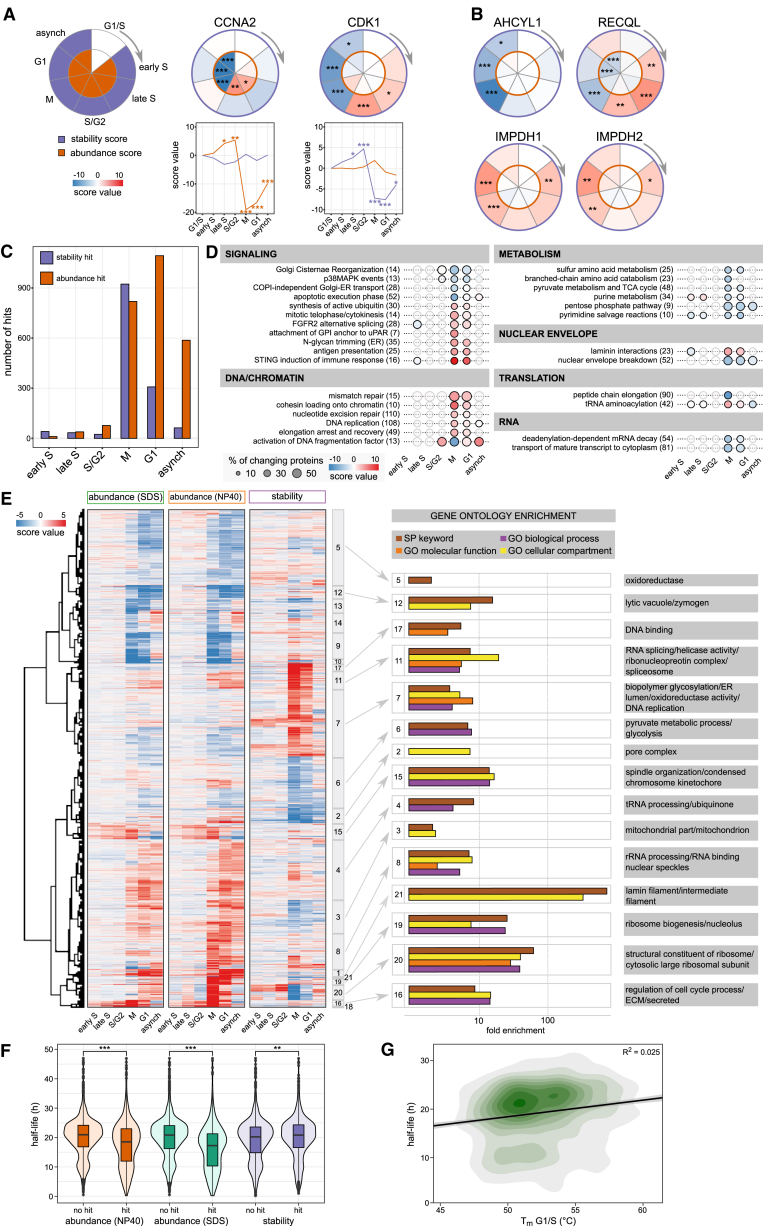
Abundance and Stability Changes of Cell-Cycle Markers, Pathways, and Cell Functions (A) Calculated abundance and stability scores for CCNA and CDK1 in a circular plot. The outer circle represents stability and the inner circle abundance. All data shown in the paper represent three independent biological replicates. ^∗^p < 0.05, ^∗∗^p < 0.01, and ^∗∗∗^p < 0.001, unless otherwise indicated. (B) Cell-cycle-dependent changes in protein stability of AHCYL1, RECQL, IMPDH1, and IMPDH2. (C) Number of significantly affected proteins in either stability (purple) or abundance (orange). (D) Pathways from the Reactome Pathway Database indicate how many proteins are associated with the pathway in general (number in brackets); the percentage of proteins affected in the pathway in at least one cell-cycle stage is correlated with the size of the circle. The color of the circle indicates the mean stability of the pathway components that significantly change in their stability. If the circle is fully opaque, the adjusted combined p value is < 0.05; if the bubble is transparent, then proteins were quantified but did not change in their stability in the respective cell-cycle stage. Pathways are categorized into broader functional groups. (E) Clustering stability and abundance of significantly changing proteins results in 21 individual clusters that were analyzed using DAVID (https://david-d.ncifcrf.gov/) with all quantified proteins as a background. The top result (lowest FDR) for all functional categories (colored as indicated in the legend) is shown for each cluster for which the FDR < 0.05 and linked to the heatmap. (F) Violin plots with the indicated median and interquartile range comparing protein half-life (from Boisvert et al. [2012]) distributions of proteins changing significantly in abundance or stability compared with non-changing proteins. Significance levels obtained from a Wilcoxon signed-rank test were encoded as ^∗^p < 0.05, ^∗∗^p < 0.01, and ^∗∗∗^p < 0.001. (G) 2D density plot of protein half-lives comparison with protein melting points in G1/S phase fitted by a linear model. See also Figures S1 and S2 and Tables S2, S3, S4, and S5.

To derive changes in protein thermal stability and abundance across cell-cycle stages, we used two-dimensional TPP (2D-TPP), which multiplexes different cell-cycle stages at each temperature (Becher et al., 2016) (see Figure 1; STAR Methods). This analysis yields protein abundance ratios to the reference condition, G1/S phase. We obtained quantitative data across the different cell-cycle stages and temperatures for each of the 4,970 proteins that passed our quality control requirements (see STAR Methods), out of the total set of 10,064 identified proteins (Table S1). We calculated abundance and stability scores for each protein and determined the significance based on three biological replicates (see Figures 1 and 2A; Table S2; STAR Methods).

Changes in protein abundance can reflect altered protein levels or altered solubility, with the latter capturing e.g., a transition from a polymerized to a depolymerized state. To disentangle these two possibilities, we also quantified the total amount of protein in the cell after solubilizing with a strong detergent (SDS) (Table S3).

To capture the shape of protein melting curves, we applied the TPP temperature range (TPP-TR) approach to mitotic and G1/S cells (Savitski et al., 2014); doing so multiplexes ten different temperatures for each stage (Figure 1; Table S4). Hereafter, we will use the term “stability” to refer to the thermal stability of proteins, i.e., a protein becomes more stable if exposure to a higher temperature is required in order to cause this protein to aggregate inside the cell. We will use the term “solubility” to refer to differences in the observed abundance of a protein in mild (NP40) versus strong (SDS) detergent.

Overall, we generated a comprehensive catalog of protein abundance, stability, and solubility, with detailed temporal cell-cycle resolution.

### Known Cell-Cycle Factors Show Changes in Stability

We explored our datasets for well-characterized cell-cycle factors. For instance, cyclin-A2 (CCNA2) varied in abundance, but not in stability (Figures 2A and S1E). In contrast, cyclin-dependent kinase 1 (CDK1) did not show significant changes in abundance across the cell cycle (Malumbres and Barbacid, 2009), but its thermal stability dramatically declined after G2 (Figures 2A and S1E).

Strikingly, several proteins with functions relevant to the cell cycle displayed altered stability rather than abundance during the cell cycle (Figure 2B). Among these, we identified S-adenosylhomocysteine hydrolase-like protein 1 (AHCYL1), which inhibits ribonucleotide reductase large subunit (RRM1) in a cell-cycle-dependent manner (Arnaoutov and Dasso, 2014); the ATP-dependent DNA helicase Q1 (RECQL); and inosine-5′-monophosphate dehydrogenase 1 and 2 (IMPDH1, IMPDH2), which maintain the intracellular balance of A and G nucleotides and thus play a key role in cell proliferation. Consequently, the stability changes of other proteins so far not implicated in the cell cycle should be indicative of their cell-cycle-related roles, as will be discussed and shown below.

### Protein Abundance and Stability Independently Vary from Each Other during the Cell Cycle

Overall, we uncovered significant cell-cycle-dependent changes in stability and abundance for 1,592 and 1,044 unique proteins, respectively (Figure 2C; Table S2). Most abundance changes occurred in early G1, whereas most stability changes occurred in mitosis.

**Figure S2:**
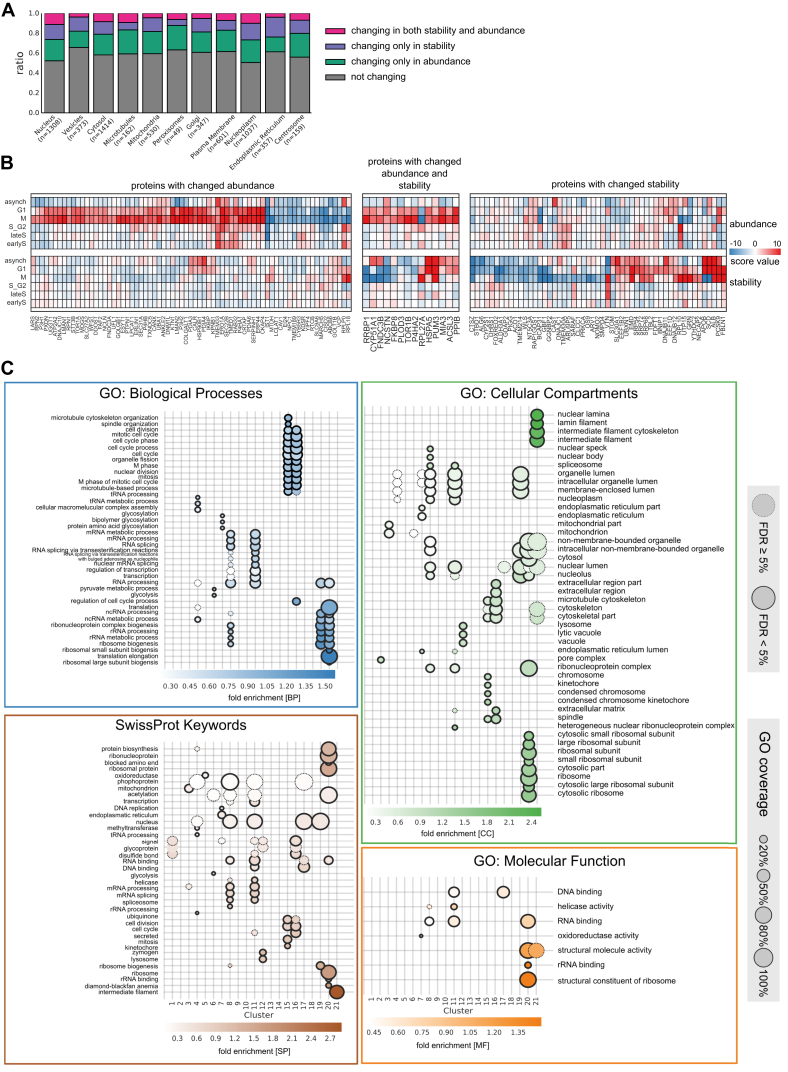
Analysis of Cell-Cycle-Dependent Stability Effects on Organelle-Specific Proteins and Fine-Grained GO Results, Related to Figure 2E (A) Bar plots showing the ratio of organelle-specific proteins (as defined from gene ontology) affected in either stability or abundance or both. (B) Heatmap showing ER-associated/localized proteins that are significantly affected in their abundance (left panel), stability (right panel) or both (middle panel) during mitosis. (C) GO-enrichment results from DAVID (https://david-d.ncifcrf.gov/) with the colors encoding different broad functional categories. The size of the bubble illustrates the relative coverage of the respective GO-category, and the color of the bubbles shows the fold enrichment, x axis with the 21 clusters defined in Figure 2E.

We used gene ontology (GO) analysis to obtain an overview of which cellular processes and substructures are most prominently affected by cell-cycle-dependent changes in stability. We identified various processes related to the cell cycle (Figure 2D; Table S5), such as nuclear envelope breakdown and the purine salvage pathway. During mitosis, we observed stabilization of signaling-, DNA-, and chromatin-associated proteins and destabilization of proteins involved in metabolic processes. The subcellular distribution of proteins displaying altered stability was relatively even (Figure S2A); however, the endoplasmic reticulum contained more proteins with variable stability (p value 3.04 × 10^−5^, Fisher’s exact test) (Figures S2A and S2B). This might reflect the morphological changes in the endoplasmic reticulum during mitosis (Schwarz and Blower, 2016).

To explore a potential relationship between stability and abundance, we clustered the combined abundance and stability scores based on their Euclidean distance (Figure 2E; Table S2). The resultant 21 distinct clusters primarily consisted of proteins affected in either their stability or their abundance (Figures 2E and S2C), strongly suggesting that these proteomic traits are controlled independently. Comparison of our relatively quantified data with previously determined protein half-lives (Boisvert et al., 2012) revealed that proteins changing in abundance across the cell cycle turn over significantly faster (Figure 2F). In contrast, proteins changing in stability tend to have longer half-lives, but with a smaller effect size (Figure 2F). Further, protein melting points were not correlated with protein half-lives (Figure 2G). We conclude that the cell-cycle-dependent variance of stability is controlled through mechanisms that do not affect protein abundance.

### Stability Changes Reveal Cell-Cycle Functions for Enzymes

**Figure 3:**
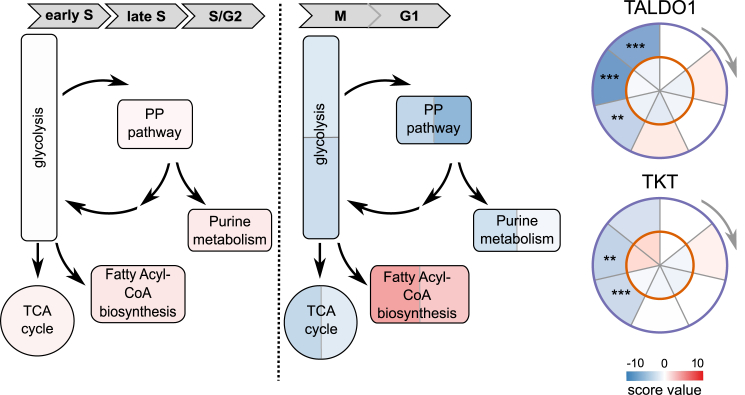
Metabolic Pathways Cell-cycle effect on the stability of protein components of the pentose phosphate pathway (PPP). On the left-hand side, the PPP is shown along with related pathways colored by average stability across the stages from early S to S/G2. On the right-hand side, the same constellation is shown. It is colored in stability values derived from mitosis (left) and G1 phase (right) for each protein. TKT and TALDO1 are two enzymes of the PPP that become significantly destabilized upon the onset of mitosis. See also Figure S3 and Table S2.

Cell-cycle progression is tightly coupled to intracellular metabolism (Lee and Finkel, 2013). However, it has been challenging to identify metabolic enzymes that are more active because of increased substrate availability, rather than increased enzyme abundance. Since substrate binding increases the stability of enzymes (Savitski et al., 2014), our data can serve as a proxy for enzyme activity. Indeed, the variation of enzyme stability coincided with cell-cycle progression for several interconnected metabolic pathways (Figure 3A; Table S5). The fatty acid biosynthesis pathway is required for mitotic exit (Scaglia et al., 2014), and its key enzymes were stabilized in mitosis and early G1. The non-oxidative branch of the pentose phosphate pathway (PPP) has an increased metabolic flux at G1/S (Fridman et al., 2013) that is reflected in the higher stability of the respective enzymes. This provides evidence for cell-cycle-dependent changes in the non-oxidative branch of the PPP not only on the metabolic but also on the proteome level.

**Figure S3:**
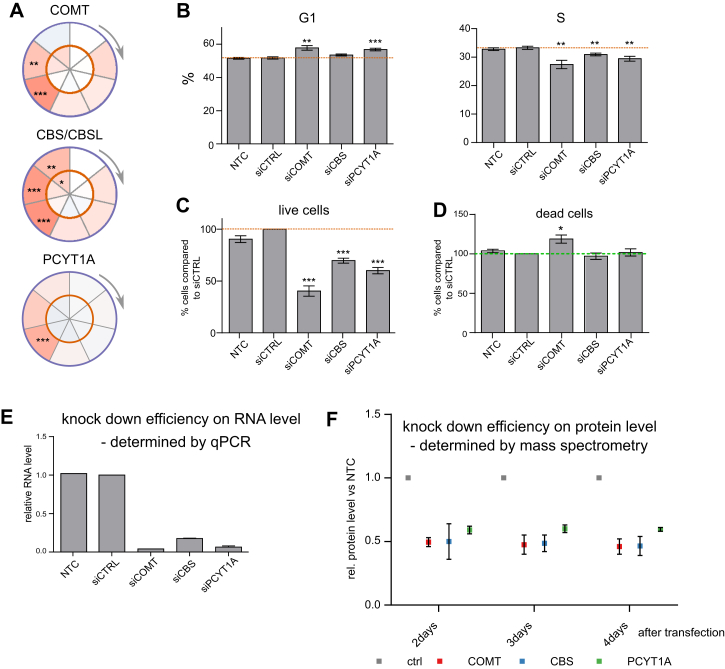
Related to Figures 3B and 3C (A) Circle plots for potential new cell cycle markers changing in their stability during the cell cycle (COMT, CBS/CBSL, PCYT1A). (B) Knock-down of the proteins shown in A results in significant changes in percentage of cells in G1 and S (assessed after 4 days knock-down by PI staining and flow cytometry). (C) Knock-down of the proteins shown in A results in significant changes in percentage of live cells (assessed after 4 days knock/down using a cytotoxicity assay) compared with siCTRL. (D) Knock-down of the proteins shown in A results in minor changes in percentage of dead cells (assessed after 4 days knock/down using a cytotoxicity assay). (E) Knock down efficiency on mRNA level (qPCR), two biological replicates. (F) Knock down efficiency on protein level (mass spectrometry-based quantification), two biological replicates. SEM is shown.

Furthermore, we depleted three metabolic enzymes with cell-cycle-dependent stability patterns: catechol-O-methyltransferase (COMT), cystathionine beta-synthase (CBS), and choline-phosphate cytidylyltransferase A, (PCYT1A). Doing so significantly reduced both the frequency of cells in S phase and proliferation (Figures S3A–3F).

In summary, our data not only recapitulate the importance of previously characterized enzymes contributing to cell-cycle progression but they also can be mined to identify the time-resolved metabolic activity of enzymes.

### RNA Polymerase II Stability Correlates with Transcriptional Activity

**Figure 4:**
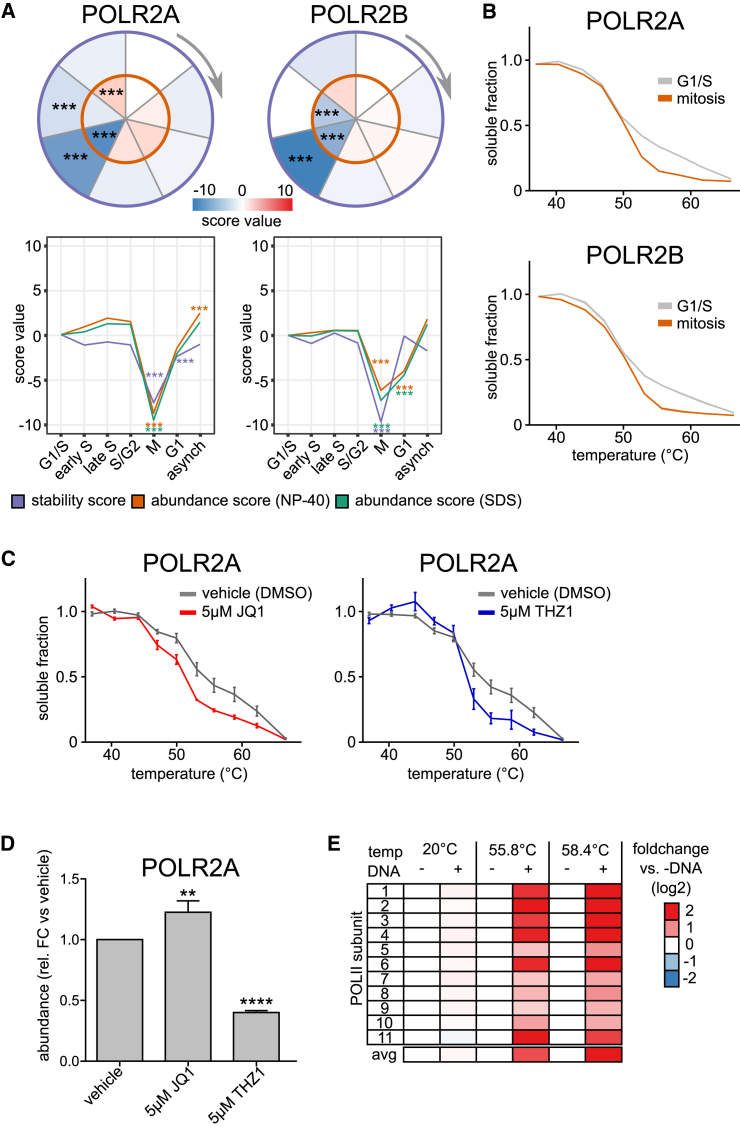
Thermal Stability and Activity of RNA Polymerase II in Mitosis (A) Circle and line plots for two subunits of RNA Pol II (POLR2A and POLR2B) show the significant change in stability (purple) and abundance (NP-40, orange, SDS green). (B) Melting curves (generated in TPP-TR experiments) for POLR2A and POLR2B show the biphasic melting behavior in G1/S, compared with mitosis. (C) TPP-TR experiment of HeLa cells treated with vehicle (DMSO), BET inhibitor JQ1, or CDK7 inhibitor THZ1. Quantified western blot data for POLR2A. SEM is shown. (D) Quantification of POLR2A protein level after a 3-hr treatment with DMSO, JQ1, or THZ1 (western blot quantification). SEM is shown. (E) Stabilization of purified RNA Pol II complex from yeast by its specific DNA sequence. The stabilization was calculated as a fold change against the control (without DNA) for each temperature. Coomassie gel quantification. See also Figure S4 and Tables S2, S3, and S4.

Next, we investigated whether our data capture changes in protein-chromatin interactions. We found that RNA polymerase II (Pol II) subunits POLR2A and POLR2B were significantly decreased in both abundance and stability in mitosis (Figure 4A). The melting profiles of POLR2A and POLR2B displayed biphasic behaviors in interphase cells, but were monophasic in mitosis (Figure 4B; Table S4), suggesting the existence of two distinct populations of RNA Pol II subunits in interphase.

**Figure S4:**
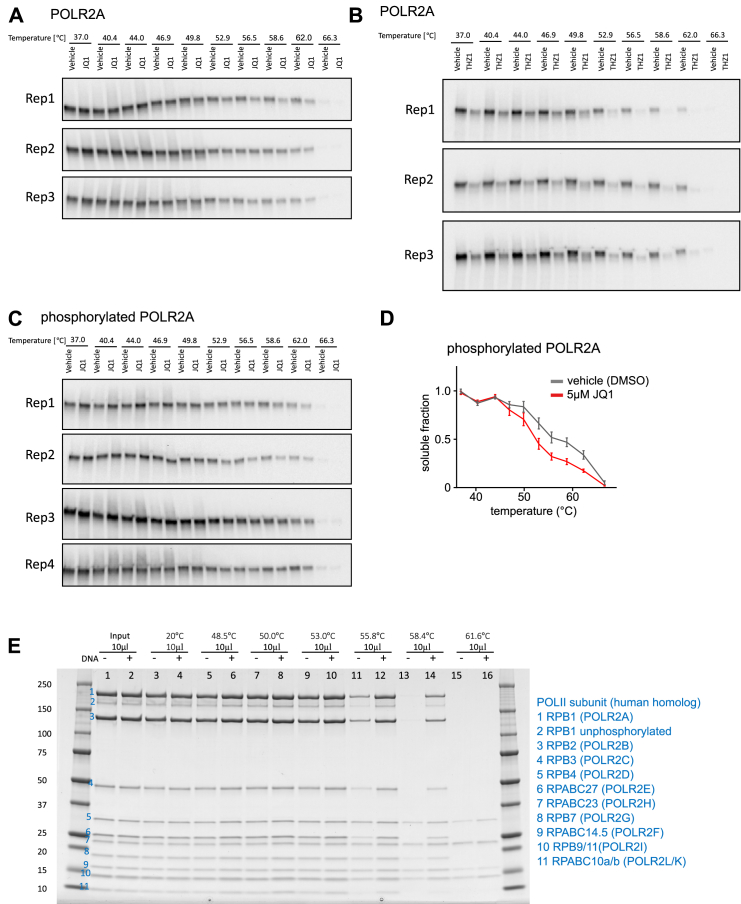
Related to Figure 4 (A) Western blot images used for quantification of POLR2A destabilization upon 3h treatment with JQ1. (B) Western blot images used for quantification of POLR2A destabilization upon 3h treatment with THZ1, as well as melting curve plot derived from this image. (C) Western blot images used for quantification of phosphorylated POLR2A destabilization upon 3h treatment with JQ1. (D) Quantification of (C). SEM is shown. (E) Coomassie gel showing purified RNA Pol II complex from yeast. From left to right with increasing temperature, always control and treatment (+DNA) next to each other. Blue numbers indicate the subunits separated on the gel.

Transcriptional arrest occurs in mitosis, and it has been reported that RNA Pol II is cleared from chromatin (Liang et al., 2015). We hypothesized that the distinct RNA Pol II populations in interphase reflect a transcriptionally inactive subpopulation not bound to DNA and a more stable, transcriptionally active subpopulation bound to DNA. To test this, we treated asynchronous cells with the bromodomain inhibitor JQ1, which causes transcription elongation defects (Lovén et al., 2013). JQ1 treatment led to reduced RNA Pol II stability (Figures 4C and S4A) and a slight increase in abundance (Figure 4D), suggesting that RNA Pol II stability correlates with transcriptional activity.

The phosphorylation state of the POLR2A carboxy-terminal domain (CTD) changes at different stages of transcription, although both phosphorylated and unphosphorylated forms occur in the subpopulation that is not bound to DNA (Komarnitsky et al., 2000). To rule out that the change in POLR2A stability is solely due to a change in phosphorylation state, we used a phospho-specific antibody to monitor POLR2A stability. JQ1 treatment destabilized phosphorylated POLR2A (Figures S4C and S4D), indicating that the reduced stability was not driven by dephosphorylation. We also treated HeLa cells with THZ1, a CDK7 inhibitor that has been reported to reduce RNA Pol II occupancy at promoters and gene bodies (Kwiatkowski et al., 2014). THZ1 treatment led to reduced POLR2A stability (Figures 4C and S4B) and reduced POLR2A levels (Figure 4D), both of which likely contribute to the potent transcriptional arrest observed with this inhibitor (Kwiatkowski et al., 2014).

To confirm that DNA-binding stabilizes RNA Pol II, we performed thermal shift experiments on purified yeast RNA Pol II in the presence of DNA. Consistent with the above observations, the addition of DNA stabilized RNA Pol II (Figures 4E and S4E). These findings suggest that the decreased stability in mitosis compared to interphase is due to the clearance of a subpopulation of RNA Pol II from chromatin and coincides with a downregulation of the overall RNA Pol II levels. In general, our results suggest that the measurement of RNA Pol II stability *in situ* correlates with the transcriptional activity in cells.

### Complex-Dependent Variation in Stability across the Cell Cycle

**Figure 5:**
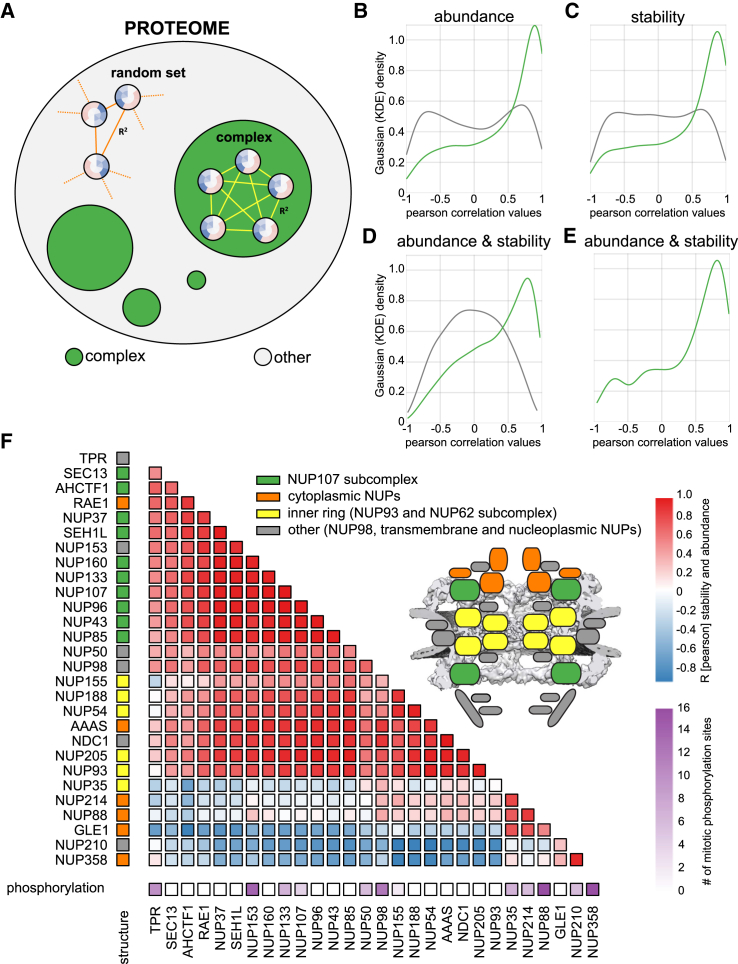
Co-stability of Known Protein Complexes and Submodules of the NPC (A) Schematic illustration of correlation analysis (see STAR Methods for further details). (B–D) Density graph of correlation coefficient values (Pearson) calculated from abundance (B), stability (C), and concatenated abundance-stability (D) profiles between proteins known to be members of the same complex (green). The gray density shows correlation values from all combinations of proteins not associated with any complex. (E) Density graph of correlation values (Pearson) calculated from concatenated abundance-stability profiles of all subunits of the nuclear pore complex (NPC). (F) Correlation matrix of NPC subunits based on their concatenated abundance-stability profiles. The colors on the left indicate their association with a specific substructure of the NPC, as colored in the respective cartoon. The scale on the bottom of the matrix indicates how many mitotic phosphorylation sites the corresponding protein subunit contains as described by Olsen et al. (2010). See also Figure S5 and Table S2.

**Figure S5:**
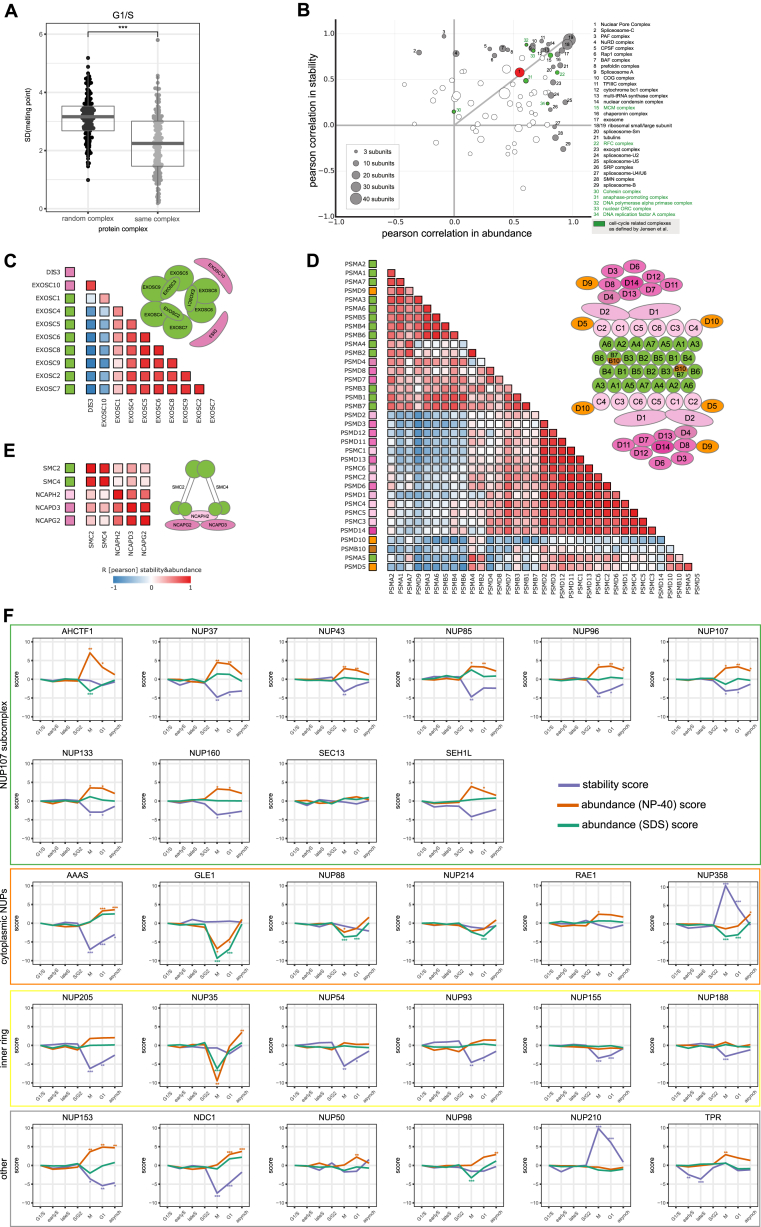
Overview of Complex Co-stability and Co-abundance and Differential Stability Pattern of Moonlighting Subunits of the Exosome Complex, Related to Figure 5 co-co-(A) Melting temperatures of same protein complexes in G1/S compared to random complex assignment. (B) Scatterplot showing complex median co-abundance (median correlation calculated from abundance profiles of corresponding subunits) versus complex median co-stability (median correlation calculated from stability profiles of corresponding subunits). (C) Exosome structure with indicated catalytic subunits, DIS3 and EXOSC10 (light red). Density graph of correlation values (Pearson) calculated from concatenated abundance-stability profiles of all subunits of the Exosome complex, as well as the corresponding correlation matrix as designed for the NPC (see Figure 6F). (D) Correlation matrix of proteasome-subunits based on their concatenated abundance-stability profiles. The colors on the left indicate their association with a specific sub-structure of the proteasome, as colored in the respective cartoon. (E) Correlation matrix of condensin II-subunits based on their concatenated abundance-stability profiles. The colors on the left indicate their association with a specific sub-structure of condensin II, as colored in the respective cartoon. (F) Line plots for all measured subunits of the NPC-complex showing stability (purple), abundance (NP-40, orange) and abundance (SDS, green) scores.

We calculated the correlation of the abundance and stability values of proteins that are subunits of the same annotated complex (Ori et al., 2016) and compared the resultant distribution to correlation values stemming from all other proteins that are not part of annotated complexes (Figure 5A). The abundance profiles of protein complex subunits across the cell cycle were highly correlated (Figure 5B), even stronger correlation was observed for stability (Figure 5C), suggesting that protein complexes mostly melt as a whole unit *in situ* once a critical temperature is reached. Indeed, protein complex subunits have a significant tendency toward coherent melting behavior (Figure S5A). Combined stability and abundance values yielded the best discrimination between proteins that are part of complexes from those that are not, including complexes with temporally regulated assembly (Jensen et al., 2006) (Figures 5D and S5B).

We found that subunit correlation patterns are indicative of stable subcomplexes, or of subunits that are loosely associated or potentially moonlight in other complexes. For example, in the exosome complex (Figure S5C) the core components displayed a high degree of correlation, while the two catalytic subunits, DIS3 and EXOSC10, did not follow this pattern. While the core subunits were collectively destabilized in mitosis, DIS3 and EXOSC10, which are uniquely required for mitotic progression in fission yeast (Murakami et al., 2007) and *Drosophila* (Graham et al., 2009), displayed increased stability in mitosis. Likewise, the 26S proteasome, a complex linked to cell-cycle progression (Guo et al., 2016), showed a different stability and abundance variation in a subset of proteins belonging to the 19S regulatory subcomplex (Figure S5D). Furthermore, we observe that the core SMC subunits of condensin II have a distinct behavior from the rest of the complex (Figure S5E).

Also in case of the nuclear pore complex (NPC), the combined nucleoporin (Nup) stability and abundance profiles clustered into known subcomplexes (Figures 5E and 5F). The NPC has a cell-cycle-dependent assembly status: it is membrane bound in interphase but resolves into soluble subcomplexes during mitosis, as triggered by phosphorylation (Laurell et al., 2011). Surprisingly, the stability behavior of the distinct subcomplexes was strikingly different, even with opposite sign. Major scaffolding subcomplexes showed a minor destabilization in mitosis, indeed mirroring the NPC assembly status. The effect sizes for the Y and inner ring complexes are slightly different, possibly reflecting architectural disparities that impact on their extractability (Figures 5F and S5F). In striking contrast, most intrinsically disordered Nups, such as NUP358 (RANBP2), remained stable in mitosis or were even strongly stabilized. This is unexpected, because many of the Nups with high intrinsically disordered protein (IDP) content are aggregation prone proteins (Lemke, 2016).

### Disordered Proteins Are Stabilized during Mitosis

NUP358 critically contributes to Y complex oligomerization in intact NPCs during interphase (von Appen et al., 2015). In mitosis, however, it dissociates from the Y complex and localizes to the spindle region (Joseph et al., 2004), and depletion of NUP358 leads to spindle defects (Hashizume et al., 2013). NUP358 is a large, multifunctional protein that contains several folded domains linked by long, intrinsically disordered regions that are important for nucleocytoplasmic exchange in interphase.

**Figure 6:**
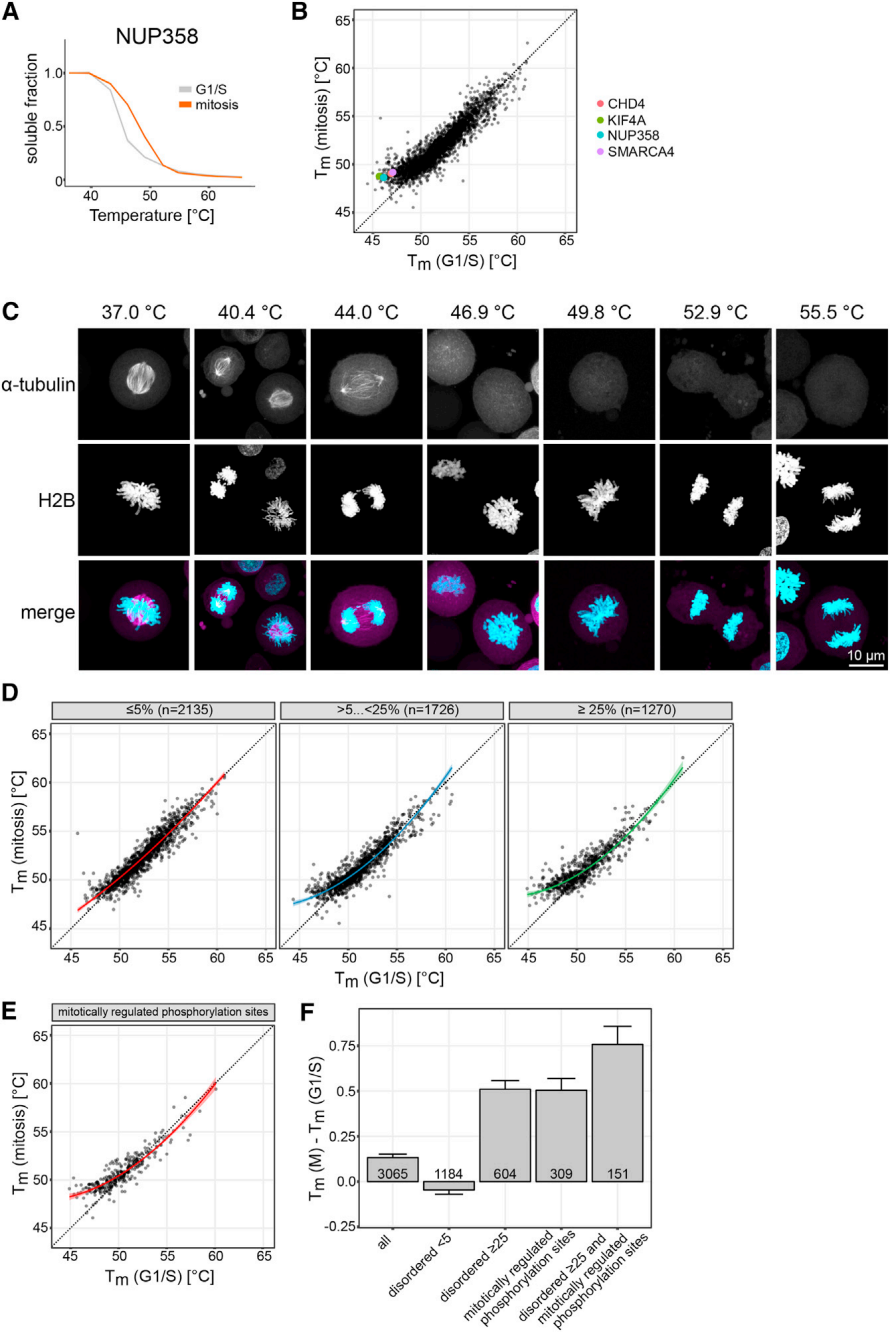
Disordered, Mitotically Phosphorylated Proteins Are Stabilized during Mitosis (A) Melting curves of NUP358 in G1/S and mitosis. Data represent three biological replicates. (B) Scatterplot comparing the melting temperatures (T_m_) for proteins in G1/S (x axis) and M (y axis). A shift toward higher melting points in mitosis for representative proteins CHD4, KIF4A, NUP358, and SMARCA4 are indicated with coloring. (C) Microscopy images of the mitotic spindle at different temperatures. Mitotic HeLa Kyoto EGFP-alpha-tubulin/H2B-mCherry cells were imaged after heat treatment. z stacks maximum intensity projections of samples treated at the indicated temperatures are shown. On the merged images, α-tubulin and H2B are shown in magenta and cyan, respectively. (D) Scatterplots illustrate the melting point shift observed from G1/S (x axis) to M (y axis) at different cutoffs for the relative coverage of disordered regions (indicated above each plot as a percentage). (E) Shift of melting point for proteins containing mitotically regulated phosphorylation sites as described in Olsen et al. (2010). (F) Melting point difference for proteins with different levels of disordered regions in their sequence and containing mitotic phosphorylation sites. SEM is shown. See also Figure S6 and Tables S4 and S6.

**Figure S6:**
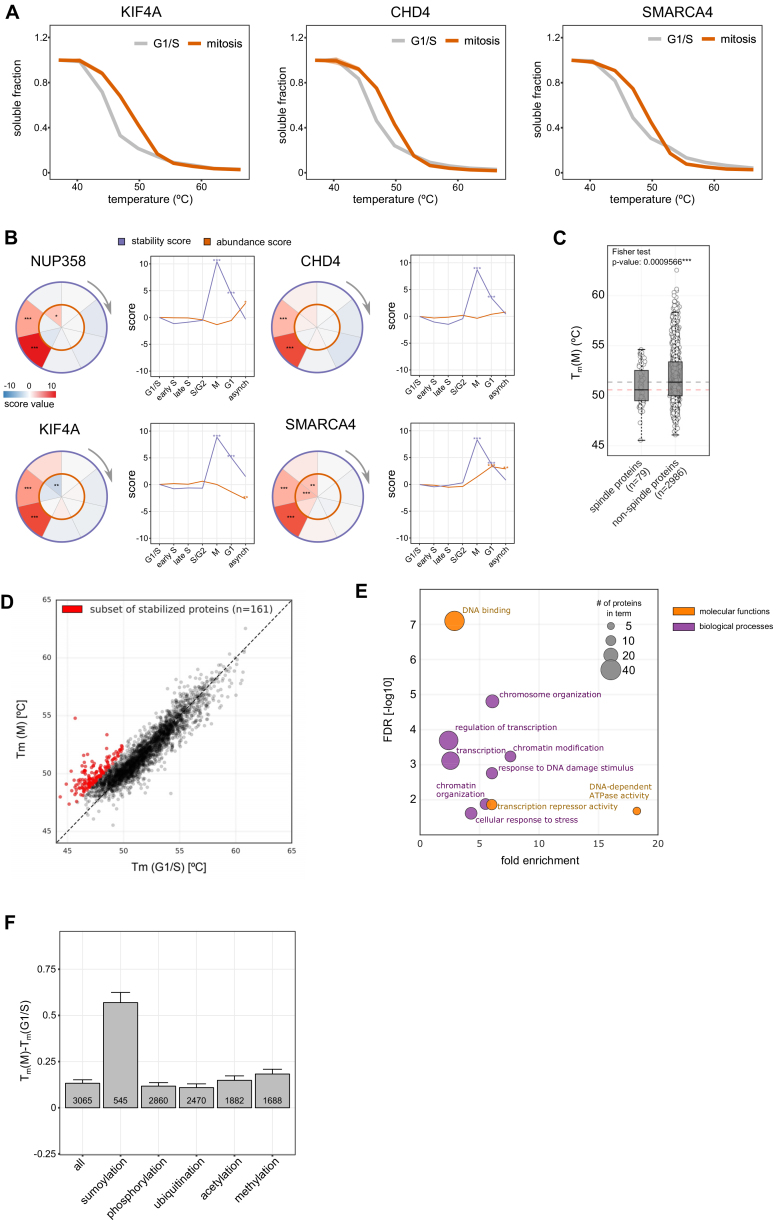
Related to Figure 6 (A) Melting curves for CHD4, KIF4A and SMARCA4A. Data shows the median for three replicates. (B) Circle and line plots showing abundance (NP-40, orange) and stability scores (purple) for NUP358, CHD4, KIF4A and SMARAC4. (C) Boxplot comparing melting temperatures in M, on the left are annotated spindle proteins from Sauer et al. The horizontal lines show the median of non-spindle proteins (black line) and spindle proteins (red line) indicating the lower melting temperatures of spindle proteins. (D) Melting temperatures for G1/S and M. Proteins in red were selected for further GO analysis shown in (E). (E) GO for spindle proteins shown in (D). GO analysis using DAVID (https://david-d.ncifcrf.gov/) was conducted on the protein set that is stabilized in mitosis relative to the G1/S reference point. For filtering we considered proteins that were significantly stabilized (p value < 0.1) and with their melting point in mitosis below 55°C and in G1/S below 50°C. The resulting 161 proteins were primarily found to be involved in DNA-binding activity and chromatin-associated processes. The scatter illustrates the fold-enrichment against the respective FDR for terms derived from the broad categories “molecular function” and “biological processes.” The size of each bubble relates to the number of proteins identified for each term. (F) Difference in melting points (G1/S versus M) of all proteins, and proteins annotated with different post-translational modifications (specified in the STAR Methods). SEM is shown.

Similarly to NUP358 (Figure 6A), several other confirmed spindle binding proteins displayed strong mitotic stabilization, such as CHD4 (Yokoyama et al., 2013), KIF4A (Kurasawa et al., 2004), and SMARCA4 (Yokoyama et al., 2009) (Figures 6B, S6A, and S6B). Compared to the entire proteome, annotated mitotic spindle binding proteins (Sauer et al., 2005) had a significantly lower stability (Figure S6C; Table S6). Since the aggregation of several of these proteins in mitosis was already substantial at 47°C (30% on average as in the case of NUP358) (Figure 6A), one would expect this to have a catastrophic effect on the spindle integrity. Indeed, heating mitotic cells to 47°C resulted in a severely damaged mitotic spindle, and at 50°C no traces of the spindle were detectable (Figure 6C).

We further examined proteins that were stabilized in mitosis and found common functional and biophysical characteristics: such proteins were significantly enriched (false discovery rate [FDR] < 5%) for the GO terms, DNA binding, chromosome and chromatin organization, and transcription-related processes (Figures S6D and S6E; Table S5). Systematic computational partitioning of proteins according to the fraction of their sequence predicted as disordered (Oates et al., 2013) revealed that the mitotic stabilization behavior is specific to proteins with large proportions of disordered regions (Figure 6D).

The synchronized stability change of the disordered proteins during mitosis suggests the presence of a regulatory switch. We considered mitotic phosphorylation, given that it is known to occur in disordered regions (Tyanova et al., 2013), and that phosphorylation-mediated folding of disordered proteins has been reported (Bah et al., 2015). We found that proteins with annotated mitotic phosphorylation sites (Olsen et al., 2010) (Table S6) were stabilized in mitosis, and that there is a considerable overlap of disordered and mitotically phosphorylated proteins that exhibit the strongest stabilization (Figures 6E and 6F). A similar trend is observed for sumoylation, a modification linked to cell-cycle progression and mitosis (Pelisch et al., 2014) and crosstalk with phosphorylation (Yao et al., 2011) (Figure S6F). The trend is not observed when non-mitotic phosphorylation or ubiquitination, acetylation or methylation sites are considered (Figure S6F) (Hornbeck et al., 2015).

Taken together we observe that chromatin and spindle related proteins with low stability and disordered regions are stabilized in mitosis. This effect is most pronounced for proteins that contain annotated mitotic phosphorylation sites.

### Solubility Changes Capture Cell-Cycle-Dependent Phase Transitions

**Figure 7:**
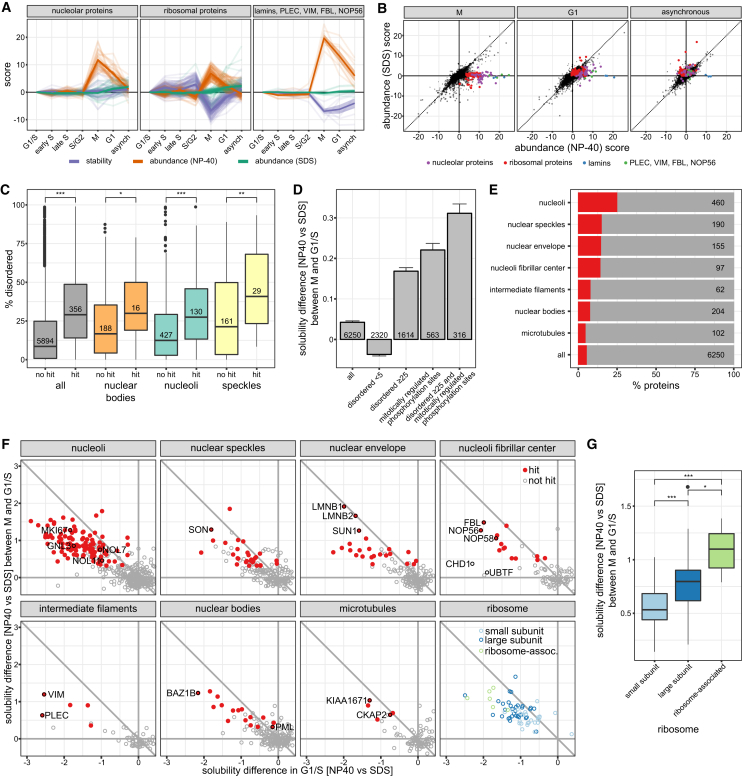
Solubility Transitioning Proteome (A) Abundance (measured in NP-40 lysis buffer in orange and SDS lysis buffer in green) and stability (purple) profiles of three protein groups (nucleolar, ribosomal, or lamins and others from cluster 19, 20, or 21 as shown in Figure 2E, respectively) are shown across all cell-cycle stages. (B) Scatterplot depicting the abundance scores in SDS lysis (y axis) versus abundance scores from NP-40 lysis (x axis) for each protein. The indicated functional groups are solubilized during mitosis, but not in G1, except for lamins, which also remain soluble during G1 phase. (C) Boxplots of relative coverage of disordered regions for proteins significantly changing in solubility in mitosis versus G1/S compared to other proteins. Comparisons are made for all proteins, as well as for proteins located within different membraneless organelles as defined by the Human Protein Atlas (HPA). (D) Solubility differences in mitosis versus G1/S for proteins with different levels of disordered regions in their sequence and containing mitotic phosphorylation sites. SEM is shown. (E) Percentage of the proteomes of different organelles significantly changing in solubility in mitosis versus G1/S and localization as defined by HPA, except for the nuclear envelope, which combines nuclear membrane annotated proteins as defined by HPA and proteins annotated as inner nuclear membrane proteins by Wilkie et al. (2011). (F) Scatterplots comparing the solubility of proteins in G1/S and the relative change in solubility of proteins in mitosis versus G1/S for different organelles, the cytoplasmic ribosomal subunits, and the ribosome-associated complex determined by Havugimana et al. (2012) (BOP1, RRS1, GNL3, EBNA1BP2, FTSJ3, MKI67). Proteins with negative x axis values and close to zero y axis values are insoluble in G1/S and remain insoluble in mitosis. Proteins with negative x axis values and positive y axis values are insoluble in G1/S and become more soluble in mitosis. Proteins that are significantly more soluble in mitosis compared to G1/S are marked in red. (G) Solubility differences in mitosis versus G1/S compared between proteins from the small and large cytoplasmic ribosomal subunits and the ribosome associated complex determined by Havugimana et al. (2012). See also Figure S7 and Tables S3 and S7.

The control measurements of protein abundance in mild and strong detergent that we had originally introduced to disentangle protein expression from solubility, indicated striking solubility transitions in well-defined subproteomes at specific cell-cycle stages. For example, ribosomal proteins, nuclear lamins, vimentin and plectin, all displayed a strong transition from a non-soluble to soluble state in mitosis (clusters 19, 20, and 21; Figures 2E, 7A, and S2). This observation reflects known properties of cellular substructures: nuclear lamins and vimentin are both phosphorylated, leading to depolymerization of filaments in mitosis, and plectin undergoes a phosphorylation-triggered transition from an insoluble to soluble state (Chou et al., 1990, Foisner et al., 1996). Strikingly, the nuclear lamins showed a delay in the transition back to the insoluble state in early G1 (Figures 7A and 7B).

**Figure S7:**
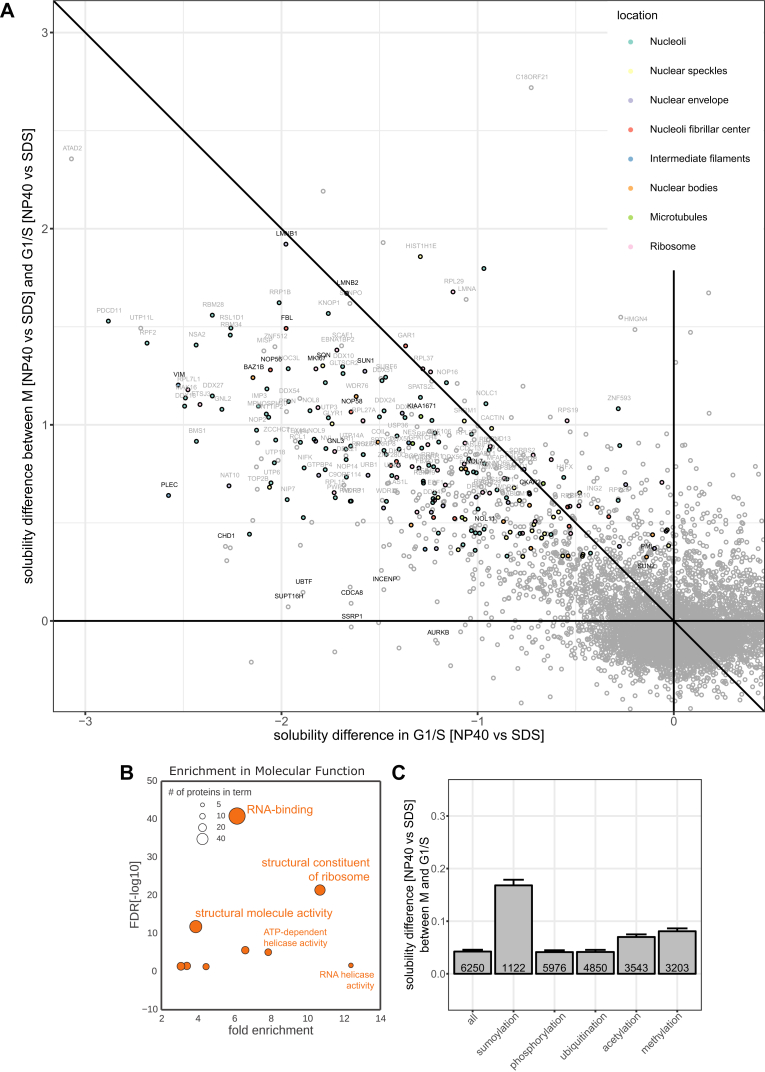
Related to Figure 7 (A) Scatterplot comparing the solubility of proteins in G1/S and the relative change in solubility of proteins in mitosis versus G1/S. Proteins with negative x axis values and close to zero y axis values are insoluble in G1/S and remain insoluble in mitosis. Proteins with negative x axis values and positive y axis values are insoluble in G1/S and become more soluble in mitosis. (B) GO analysis using DAVID (https://david-d.ncifcrf.gov/) was conducted on the protein set that is significantly more soluble in mitosis versus G1/S. The scatter illustrates the fold-enrichment against the respective FDR for terms derived from the broad category “molecular function.” The size of each bubble relates to the number of proteins identified for each term. (C) Solubility differences in mitosis versus G1/S of all proteins, and proteins annotated with different post-translational modifications (specified in the STAR Methods). SEM is shown.

Further, we observed that such solubility transitions captured components of phase-separated, membraneless organelles that are dissolved in mitosis. For instance, various nucleolar proteins are more soluble in mitosis when the nucleolus is dissolved (Hernandez-Verdun, 2011) (Figure 7A). Our above-described data capture such transitions only if they occur during the cell cycle, but they are not suitable to quantify the fractions of a protein in the soluble and insoluble state. Therefore, we quantified proteome solubility under mild and strong detergent conditions for the G1/S stage as compared to mitosis in a dedicated TMT experiment (Figure S7A; Table S7).

The analysis revealed that a small chromatin-associated fraction of the proteome is consistently insoluble in G1/S and mitosis. This fraction includes CDCA8, INCENP, and AURKB, which are core members of the chromosomal passenger complex (Figure S7A) and key regulators of several mitotic events (Carmena et al., 2012), and SUPT16H and SSRP1 of the FACT complex, which is essential for microtubule growth and bundling in mitosis (Zeng et al., 2010) (Table S7). We did not observe a single protein becoming more than 2-fold less soluble in mitosis compared to G1/S (Table S7), indicating that there is no pronounced transition from a soluble to insoluble state upon mitotic entry. In striking contrast, various proteins became significantly more soluble in mitosis. Similarly, but not identical, to the mitotically stabilized IDP proteome discussed above, those proteins were strongly enriched in RNA binding functions (Figure S7B), mitotic phosphorylation sites (Olsen et al., 2010) (Figures 7C and 7D; Table S6) and sumoylation (Figure S7C). Many of them were components of the nuclear envelope (Thul et al., 2017, Wilkie et al., 2011) and also membraneless organelles (Thul et al., 2017).

The proteins identified to undergo a solubility transition in mitosis comprise only a fraction of the previously annotated proteomes of these membraneless organelles (Thul et al., 2017), indicating that their core proteome might be less extensive than previously thought (Figures 7E and 7F). However, the subproteomes that did undergo a solubility transition were strongly enriched in disordered regions (Figure 7C) and captured key phase separating proteins of membraneless organelles. Among those were proteins with reported roles for nucleolar morphology, such as nucleostemin, GNL3 (Romanova et al., 2009), fibrillarin, FBL (Tiku et al., 2016), nucleolar protein 7, NOL7 (Kinor and Shav-Tal, 2011), and the protein SON, one of the most strongly transitioning proteins in nuclear speckles and recently proposed to be a key scaffolding factor for these membraneless organelles (Sharma et al., 2010) (Figure 7F). An exception to this trend was the transcription factor 1, UBTF (Figures 7F and S7A), which acts as a molecular beacon to recruit additional proteins during the *de novo* biogenesis of the nucleolus (Grob et al., 2014). Our data show that UBTF has a very rare behavior among the nucleolar proteins, in that it remains insoluble during mitosis.

The nucleolus is the site for ribosome assembly (Kressler et al., 2010). When it dissolves in mitosis, non and partially assembled ribosomal proteins are set free and their exact fate remains understudied. The stability of many ribosomal proteins was significantly decreased in mitosis (Figure 7A). A subpopulation of ribosomal proteins that is insoluble in G1/S becomes soluble in mitosis (Figure 7F) and might be attributed to non-assembled species. In line with this view, the insoluble interphase subpopulations of the 40S and 60S components have significantly different effect sizes (Figures 7F and 7G), reflecting the shorter residence time of 40S proteins in the nucleoulus as compared to 60S proteins (Lam et al., 2007). The ribosomal associated complex that participates in maturation of the large 60S ribosomal subunit, as determined by Havugimana et al. (2012) (BOP1, RRS1, GNL3, EBNA1BP2, FTSJ3, and MKI67), exhibited the largest effect size in its coherent solubility transition (Figures 7F and 7G), indicative of a predominant nucleolar localization in G1/S.

Taken together, our data delineate the insoluble and the cell-cycle-dependent, solubility transitioning proteome. Our experimental strategy enables unbiased assessment of key phase transitioning proteins responsible for the altered solubility state of the mitotic cell and provides molecular insight into the mitosis specific morphological changes.

## Discussion

We have performed an in-depth, proteome-wide stability profiling of the eukaryotic cell cycle. We discovered pervasive protein thermal stability variation during the cell cycle, representing a substantial layer of proteome organization that is on par with the variability of protein abundance.

Our results with RNA Pol II suggest that relative changes in transcriptional activity can be assessed *in situ* by measuring the stability of POLR2A. The disappearance of the more stable POLR2A population during mitosis coincides with the overall reduction of POLR2A levels. A speculative interpretation could be that the stable DNA-bound RNA Pol II fraction is specifically degraded upon mitotic entry.

The combined stability and abundance variation of NPC and other complexes across the cell cycle serves in delineating major subcomplexes and moonlighting subunits. This highlights the utility of the present dataset for structural biologists, as it can be used for hypothesis generation of cell-cycle-dependent complex composition and subunit function.

Mitotically phosphorylated IDPs are stabilized in mitosis. The NPC illustrates the significance of this finding. Although intrinsically disordered Nups are notoriously aggregation prone, scaffold Nups provide a grafting surface that restrains their local concentration in interphase (Lemke, 2016, Milles et al., 2013). NPC disassembly, however, does not render intrinsically disordered Nups aggregation prone. Strikingly, NUP358 that facilitates scaffold Nup oligomerization in interphase (von Appen et al., 2015) is stabilized in mitosis. These insights exemplify the challenge that cells have to deal with during the morphological changes occurring in mitosis and apparently address by major transitions in their biophysical environment. It is possible that IDPs are integrated in specific protein complexes or subcellular structures in interphase that restrain their local concentration, such is the case, for instance, for NUP358 and the NPC scaffold. In mitosis, these restraints on local concentration could potentially disappear and render IDPs aggregation prone. Phosphorylation of these proteins might be important to prevent aggregation in mitosis. Their similar biophysical milieu might phase separate them into the spindle region, where they are less exposed to aggregation triggers, such as membranes (Jiang et al., 2015).

We also mapped the subset of the proteome undergoing solubility transitions, from an insoluble to a soluble state as well as the proteins that are permanently insoluble, during the cell cycle. The molecular signature of RNA binding enriched proteins that are solubilized in mitosis is very similar to the DNA binding enriched subproteome that gets stabilized in mitosis. The two subproteomes have an overlap of 15% and are enriched in disordered regions and mitotic phosphorylation. The solubility transition of the cell in mitosis is often obvious on a morphological scale, e.g., the dissolution of the nucleolus is apparent by light microscopy. Our data categorize the proteins involved in this process, several of which play key roles in the architecture of membraneless organelles. The vast majority of all proteins that are insoluble during interphase are solubilized in mitosis. We conclude that the mitotic cell is more soluble and less aggregation prone as compared to the interphase cell.

In conclusion, our study reveals that the biophysical properties of proteins extensively vary across the cell cycle, reflecting changes in metabolic activity, complex composition, and transcriptional activity. It can thus be argued that changes in stability represent a robust way to identify proteins whose activity and interaction patterns vary across the cell cycle. In contrast, changes in abundance will typically not reflect such variations. We show the utility of our dataset for biological hypothesis generation and expect it to become widely used in metabolomics, transcription, structural biology, IDPs, and cell-cycle-related studies.

## STAR★Methods

### Key Resources Table

**Table:** 

REAGENT or RESOURCE	SOURCE	IDENTIFIER
**Antibodies**		

Phospho-Histone H3 (Ser10) Antibody (Alexa Fluor® 647 Conjugate)	Cell Signaling Technologies	#9716
PCNA (PC10) Mouse mAb (Alexa Fluor® 488 Conjugate)	Cell Signaling Technologies	#8580
Phospho-Rpb1 CTD (Ser2/5) Antibody	Cell Signaling Technologies	#4735
Rpb1 CTD (4H8) Mouse mAb	Cell Signaling Technologies	#2629

**Chemicals, Peptides, and Recombinant Proteins**		

THZ1	Cayman Chemical	9002215; CAS 1604810-83-4
JQ1	Sigma Aldrich	SML0974 Sigma; CAS 1268524-69-1
Nocodazole	Merck	487928; CAS 31430-18-9
thymidine	Merck	6060; CAS 50-89-5
POLII complex	Kireeva et al., 2003	n/a

**Critical Commercial Assays**		

MultiTox-Fluor Multiplex Cytotoxicity Assay	Promega	G9201
TMT10plex Isobaric Label Reagent Set, 3 × 0.8mg	Thermo Fisher	90111

**Deposited Data**		

Raw mass spectrometry data	Pride	PXD008646
Supplemental datasets and Complex clustering plots	Mendeley	https://doi.org/10.17632/xrbmvv5srs.2

**Experimental Models: Cell Lines**		

HeLa Kyoto	S. Narumiya	RRID:CVCL_1922
HeLa Kyoto EGFP-alpha-tubulin/H2B-mCherry	Daniel w. Gerlich	RRID:CVCL_L802

**Oligonucleotides**		

Primer COMT:	This paper, Sigma Aldrich	n/a
For: GCGACAAGAAAGGCAAGATCG		
Rev: TCCTTCACGCCAGCGAAAT		
Primer CBS:	This paper, Sigma Aldrich	n/a
For: GGGTCCCCAGAGGATAAGGAA		
Rev: ATTTTTGGAGATTTTGCCGGGG		
Primer PCYT1A:	This paper, Sigma Aldrich	n/a
For: ATGAGGTGGTGAGGAATGCG		
Rev: AACATGCCTGCCTCCTTGAT		
siRNA target COMT	Ambion	ID# s39
siRNA target CBS	Ambion	ID# s290
siRNA target PCYT1A	Ambion	ID# s10166
POLII RNA	Eurofins MWG Synthesis GmbH	n/a
5′-UAUAUGCAUAAAGACCAGGC-3′		

**Recombinant DNA**		

POLII experiments:	Eurofins MWG Synthesis GmbH	n/a
5′-AAGTCAAGTACTTACGCCTGGTCATTACTAGTACTGCC-3′		
5′-GGCAGTACTAGTAAACTAGTATTGAAAGTACTTGACTT-3′		
**Software and Algorithms**		

Isobarquant	Franken et al., 2015	https://github.com/protcode/isob
Mascot search engine	Matrix Science	http://www.matrixscience.com/
R	R Core Team	https://www.R-project.org
Resource website for data analysis	This paper, Github	https://github.com/fstein/cellcycle_TPP
Graphpad Prism 7	GraphPad Software	https://www.graphpad.com/
Python 2.7	Python Software Foundation. Python Language Reference, version 2.7	https://www.python.org/
DAVID Bioinformatics Resources 6.7	DAVID, NCI	https://david-d.ncifcrf.gov/

**Other**		

Reactome Pathway Database	Reactome	https://reactome.org/
Complex Database	Ori et al., 2016	http://www.bork.embl.de/Docu/variable_complexes/
Cyclebase 3.0	Alberto Santos, Rasmus Wernersson, Lars Juhl Jensen	https://cyclebase.org/CyclebaseSearch
The Human Protein Atlas	Thul et al., 2017	http://www.proteinatlas.org/
Database of Disordered Protein Predictions (D2P2)	Oates et al., 2013	http://d2p2.pro
PhosphoSitePlus	Hornbeck et al., 2015	https://www.phosphosite.org/homeAction.action

### Contact for Reagent and Resource Sharing

Further information and requests for resources and reagents should be directed to and will be fulfilled by the Lead Contact, Mikhail M. Savitski (savitski@embl.de).

### Experimental Model and Subject Details

#### Cell culture and cell cycle arrest

HeLa Kyoto cells (female) were cultured at 37°C, 5% CO_2_, in DMEM including 1mg/ml glucose, supplemented with 10% FBS, 1mM glutamine. Cells were synchronized in G1-S by a double thymidine block (addition of 2mM thymidine (Calbiochem, #6060) overnight, release cells for 8 hours, second block with 2mM thymidine overnight). After the second release cells were collected at four different time points: 0 hours (G1-S phase), 2 hours (early S phase), 4 hours (late S phase), and 6 hours (S-G2 phase). For synchronization in mitosis cells were treated overnight with 2mM thymidine, released for 4 hours and treated with 100 ng/ml nocodazole (Millipore, #487928) overnight. After 0.5 hour release cells were collected (M phase). For synchronizing cells in G1, 100 ng/ml nocodazole was added overnight, then mitotic cells were collected by shake-off and released into fresh medium, letting them grow for 4.5 hours, when only attached cells were collected. Also, an asynchronous culture was used as a control.

The arrest was assessed by flow cytometry, and at the same time thermal proteome profiling experiments were performed. An aliquot of the cells was kept to perform a SDS-lysis to determine protein expression changes. All experiments were done in biological triplicates.

### Method Details

#### Flow cytometry

Cell cycle analysis was performed by flow cytometry with two bivariate analyses: DNA/ Proliferating Cell Nuclear Antigen (PCNA) and DNA/ phosphorylated Serine 10 of histone H3 (pH3)

PCNA staining was adapted from Sasaki et al. (1993). 1x10^6^ cells were permeabilized for 10 min on ice with 0.1% Triton X-100/1% bovine serum albumin (BSA)/PBS, and then fixed with methanol at −20°C for 3 min. After pelleting, cells were resuspended and stored in PBS at 4°C. For pH3 staining, 2x10^6^ cells were fixed with 1% formaldehyde for 15 min at RT. Cells were then resuspended in PBS and stored in 70% ethanol at 4°C.

Both samples were spun and the cell pellet was resuspended and incubated for 15 min in PBS at room temperature before continuing with blocking in 1% BSA in PBS for 10 min. Cells were then incubated with primary antibodies (anti-PCNA PC10 conjugated with Alexa Fluor 488, Cell Signaling Technology #8580 or anti-Phospho-Histone H3 (Ser10) conjugated with Alexa Fluor 647, Cell Signaling Technology #9716S) for 1 hour at room temperature. Cells were washed and DNA was stained by addition of 20 μg/ml propidium iodide (PI) in 0.1% Triton X-100 containing 0.2 mg/ml RNase.

Samples were analyzed on a BD LSR Fortessa instrument. For PI detection, the laser line of 561 nm and 75 mV with a band pass (BP) filter of 610/20nm was used. Alexa Flour 488 was detected with a laser line of 488 nm, 50 mW, and a BP 530/30 nm. In the case of Alexa Fluor 647, a laser line of 640 nm, 80 mV and BP 670/14 was used. Cell cycle stage analysis was performed with FlowJo v10 software. Bivariate analysis of PCNA versus DNA content presented an inverted U shape; cells with the higher signal were considered to be in S-phase. Cells with a G2/M DNA content, and positive for pH3 antibody were considered mitotic. The % of cells in G1 was calculated by fitting the PI signal to a cell cycle distribution using the Watson pragmatic model approach.

#### Thermal protein profiling and sample preparation for MS

Thermal protein profiling was done as previously described (Becher et al., 2016). In brief, cells were harvested, washed with PBS and 10 aliquots, each of 0.5 × 10^6^ cells, were distributed in PCR tubes. Each aliquot was heated for three minutes to a different temperature (37.0-40.4-44.0-46.9-49.8-52.9-55.5-58.6-62.0-66.3°C). Lysis buffer (final concentration 0.8% NP-40, 1.5mM MgCl2, protease inhibitor, phosphatase inhibitor, 0.4 U/μl benzonase) was added and cells were incubated at 4°C for one hour. Protein aggregates were removed. From the soluble fraction the protein concentration was determined and for sample preparation 10 μg protein (based on two lowest temperatures) were taken further. Proteins were reduced, alkylated and digested with trypsin/Lys-C with the SP3 protocol (Hughes et al., 2014). Peptides were labeled with TMT10plex (Thermo, #90110). Here, samples were combined in two ways: either all temperatures of one cell cycle arrest were combined allowing the determination of melting curves, or all cell cycle arrests were combined for each temperature resulting in seven conditions analyzed together. Pooled samples were fractionated on a reversed phase C18 system running under high pH conditions, resulting in twelve fractions (Hughes et al., 2014). In addition, both samples from NP-40 lysis (37°C) and SDS lysis from G1/S or M arrested samples were combined, allowing the determination of protein solubility differences.

#### LC-MS/MS measurement

Peptides were separated using an UltiMate 3000 RSLC nano LC system (Thermo Fisher Scientific) equipped with a trapping cartridge (Precolumn C18 PepMap 100, 5μm, 300 μm i.d. x 5 mm, 100 Å) and an analytical column (Acclaim PepMap 100, 75 μm x 50 cm C_18_, 3 μm, 100 Å). The LC system was directly coupled to a Q Exactive Plus mass spectrometer (Thermo Fisher Scientific) using a Nanospray-Flex ion source. Solvent A was 99.9% LC-MS grade water (Fisher Scientific) with 0.1% formic acid and solvent “B” was 99.9% LC-MS grade acetonitrile (Fisher Scientific) with 0.1% formic acid. Peptides were loaded onto the trapping cartridge using a flow of 30 μL/min of solvent A for 3 min. Peptide elution was afterward performed with a constant flow of 0.3 μL/min using a total gradient time of 120 min. During the elution step, the percentage of solvent B was increased stepwise: 2% to 4% B in 4 min, from 4% to 8% in 2 min, 8% to 28% in 96 min, and from 28% to 40% in another 10 min. A column cleaning step using 80% B for 3 min was applied before the system was set again to its initial conditions (2% B) for re-equilibration for 10 minutes.

The peptides were introduced into the mass spectrometer Q Exactive Plus (Thermo Fisher Scientific) via a Pico-Tip Emitter 360 μm OD x 20 μm ID; 10 μm tip (New Objective). A spray voltage of 2.3 kV was applied and the mass spectrometer was operated in positive ion mode. The capillary temperature was 320°C. Full scan MS spectra with a mass range of 375-1200 *m/z* were acquired in profile mode in the Orbitrap using a resolution of 70,000. The filling time was maximum 250 ms and/or a maximum of 3e6 ions (automatic gain control, AGC) was collected. The instrument was cycling between MS and MS/MS acquisition in a data-dependent mode and consecutively fragmenting the Top 10 peaks of the MS scan. MS/MS spectra were acquired in profile mode in the Orbitrap with a resolution of 35,000, a maximum fill time of 120 ms and an AGC target of 2e5 ions. The quadrupole isolation window was set to 1.0 *m/z* and the first mass was fixed to 100 *m/z*. Normalized collision energy was 32 and the minimum AGC trigger was 2e2 ions (intensity threshold 1e3). Dynamic exclusion was applied and set to 30 s. The peptide match algorithm was set to ‘preferred’ and charge states ‘unassigned’, 1, 5 - 8 were excluded.

#### RNAi experiments

For knockdown experiments HeLa Kyoto cells were transiently transfected using siRNA spotted cell culture plates and CELLviewTM slides following a previously described (Erfle et al., 2007) reverse transfection protocol using pre-designed Silencer® Select siRNAs (Ambion). In brief, the transfection solution composed of 20 μl siRNA (3 μM), 7 μl Lipofectamine 2000 (Invitrogen), 12 μl Opti-MEM (Invitrogen) containing 0.4 M sucrose, and 12 μl water was prepared. After incubation for 20 min at room temperature, 29 μl of 0.2% gelatin and 3.5x10%–4% fibronectin were added. Finally, the transfection cocktail was diluted 1:50 and 300 μl were spotted on 24-well cell culture plates, or 50 μl on CELLviewTM slides respectively, followed by immediate lyophilization using the concentrator miVAC (Genevac). Dependent on the incubation time, 12500 (2d), 6250 (3d) or 3125 (4d) cells/well were seeded on the spotted 24-well plates. Additionally, a non-transfected control (NTC) was included for each experiment, where cells were seeded on non-spotted wells. The knockdown of target genes was validated on the level of mRNA by quantitative real-time PCR (qRT-PCR) analysis after 2d of transfection, as well as on the level of protein by LC-MS/MS analysis after 2, 3 and 4d of transfection (MS analysis as described before).

For qRT-PCR analysis, mRNA extraction was performed using the RNeasy Mini Kit (QIAGEN) according to the manufacturer’s instructions and including an on-column DNase I digestion. Subsequently, 700 ng total RNA were converted into cDNA using the SuperScriptTM III Reverse Transcriptase (Invitrogen) following the manual for first-strand cDNA synthesis. qRT-PCR reactions were performed on a StepOnePlus Real-Time PCR System (Applied Biosystems) using 5 ng cDNA, 0.2 μM gene-specific primers and 1x Power SYBR® Green PCR Master Mix (Life Technologies). For each condition technical triplicates were prepared and the mean threshold cycle (CT) calculated. For normalization, ACTB levels were used as an internal reference and mRNA levels relative to siCTRL were calculated according to the 2-ΔΔCt method.

**Table:** 

Target gene	Silencer® Select siRNA ID (Ambion)	Primer (5′ to 3′)
COMT	s39	For: GCGACAAGAAAGGCAAGATCG
		Rev: TCCTTCACGCCAGCGAAAT
CBS	s290	For: GGGTCCCCAGAGGATAAGGAA
		Rev: ATTTTTGGAGATTTTGCCGGGG
PCYT1A	S10166	For: ATGAGGTGGTGAGGAATGCG
		Rev: AACATGCCTGCCTCCTTGAT
siCTRL (XWNEG9)	S444246	-

Cell cycle analysis was performed by flow cytometry using PI staining. After 4d of transfection cells were harvested, washed and fixed in 66% Ethanol at 4°C for several hours. After washing, DNA was stained with PI and the cell cycle analyzed as described above. In addition, apoptosis was measured using the Annexin V-FITC Detection kit (Promokine) according to the manual.

For assessment of live and dead cells a commercial assay was used (Promega, MultiTox-Fluor Multiplex Cytotoxicity Assay) and the manufacturer’s protocol was followed.

#### CETSA with two inhibitors

HeLa Kyoto cells were treated with either 5 μM JQ1 (Sigma Aldrich), 5 μM THZ1 (Cayman chemicals) or DMSO (vehicle) for 3 h at 37°C, 5% CO_2_, in DMEM supplemented with 2% FBS and 1mM glutamine. Heat treatment, lysis and removal of aggregates were performed as described above. Abundance and melting curve of POLR2A were determined by SDS-PAGE and western blot analysis. In brief, 10 μg protein (based on the lowest temperature) were separated on 4%–15% precast polyacrylamide gels (Bio-Rad) following the manual. Blotting was performed using the Trans-Blot® Turbo™ Transfer System (Bio-Rad) according to the instructions. For detection of total and phosphorylated POLR2A, Rpb1-specific antibodies (#2629 and #4735 for Ser2/5 phosphorylation, Cell Signaling) were used and the manufacturer’s protocol followed. Chemiluminescence was recorded using the ChemiDoc imaging system (Bio-Rad) and analyzed via Image Lab 5.2.1.

#### Tubulin heatshock and microscopy

HeLa Kyoto EGFP-alpha-tubulin/H2B-mCherry (Steigemann et al., 2009) were synchronized at the G1/S transition as done for the parental cell line. After the second thymidine block, cells were released for 8 to 10 hours until most of them were in mitosis as determined by phase contrast microscopy. They were collected, washed, resuspended in PBS at 0.5 × 10^6^ cells, and subjected to a heat treatment as done for the thermal profiling and MS. Afterward they were fixed with 4% PFA for 15 min at room temperature. An aliquot of each sample was let settle down onto a well of a CELLview slide (Greiner Bio-One) previously coated with poly-L-Lys. Cells were imaged on an inverted Leica TCS SP8 STED3x microscope (Leica Microsystems, Mannheim, Germany), using a HC PL APO CS2 63x/1.40 OIL objective and a tuneable and pulsed White Light Laser (WLL, emission from 470-670nm). Standard, diffraction limited confocal images were recorded for mRFP (594 nm, BP 600-690 nm) and EGFP (488 nm, BP 495-541 nm). z stacks were acquired to cover the whole cell with a z-step size of 1 μm. Images were processed using the Fiji distribution of ImageJ (https://fiji.sc/).

#### Thermal profiling of recombinant RNAP II complex

RNAP II yeast strain was a gift from Mikhail Kashlev at the NIH/National Cancer Institute (NCI), Bethesda. Pol II was purified from *S. cerevisiae* using a His-tag purification protocol as previously described (Kireeva et al., 2003). When RNA pol II was bound to nucleic acids, then a 5-fold molar excess of a pre-annealed 38-bp transcription scaffold with a 20-nt RNA (5′-UAUAUGCAUAAAGACCAGGC-3′) as previously described (Tafur et al., 2016) was used. Briefly, the template (T) and non-template (NT) strands were mixed and annealed by heating to 95°C in RNase-free water, and then slowly cooled to 25°C in 1 hr. Then, an equimolar amount of RNA was added and annealed by incubation at RT before addition of RNA pol II.

For the thermal profiling, aliquots of purified Pol II ± DNA/RNA scaffold were incubated for 3 minutes at the indicated temperature, left at room temperature for 3 more minutes and the aggregated protein was removed by ultracentrifugation (20min at 100.000 xg at 4°C). The supernatant was used for Coomassie gel.

### Quantification and Statistical Analyses

#### MS computational analysis and normalization

Raw MS data was processed with IsobarQuant (Franken et al., 2015) and peptide and protein identification was performed with the Mascot 2.4 (Matrix Science) search engine. Data was searched against a human database from Uniprot including known contaminants and the reversed protein sequences. Search parameters: trypsin, missed cleavages 3, peptide tolerance 10ppm, 0.02Da for MS/MS tolerance. Fixed modifications were carbamidomethyl on cysteines and TMT10plex on lysine. Variable modifications included acetylation on protein N terminus, oxidation of methionine and TMT10plex on peptide N-termini. The programming language R (https://www.r-project.org/) was used to analyze the raw output data from IsobarQuant (Franken et al., 2015).

Three independent datasets were processed in total (2D-TPP data, SDS data and TPP-TR data) using a similar base workflow as described in the following: First, the output files of IsobarQuant were loaded into R and merged. The raw data was then saved in an ExpressionSet R-object. Subsequently, potential batch effects were removed using *limma* (Ritchie et al., 2015) and data was normalized using variance stabilization, *vsn* strategy (Huber et al., 2002). For each temperature, an independent normalization run was used in order to account for the decreasing intensity of signal sums with increasing temperature. To calculate the abundance and stability scores, we used a bootstrap algorithm; for calculating the expression scores, the results of the concordant limma analysis were modified. For all scores, two different false discovery rates were estimated, a local and a global FDR. The global FDR was calculated based on the score distribution (z-distribution) and thereby correlates with the effect size of the score. The local FDR, on the other hand, describes the quality and the reproducibility of the score values and takes into account the variance between replicates.

#### Thermal proteome profiling (TPP) analysis

After normalization of the raw TPP-TR data, the TPP package (Franken et al., 2015) was used to identify melting points. After normalization of the 2D-TPP dataset, fold changes were calculated against the respective 37°C and the G1/S data points. These were used for the calculation of abundance and stability scores.

For the SDS data, a *limma* analysis was performed on the normalized data by comparing each cell-cycle to the G1/S cell cycle. The resulting fold changes (logFC) were transformed into z-scores using the Cox method (Cox and Mann, 2008) in order to determine the expression scores for each protein. The global FDR for the expression score was calculated from the z-values using *fdrtool* R package taking the q-value as the output. The local FDR for the expression score was calculated by adjusting the *limma* p values for multiple testing by Benjamini & Hochberg with the *p.adjust* method (method = ”fdr”).

In order to calculate the abundance and stability scores, a bootstrap method was used with 500 iterations and a prerequisite of at least 2 measured data points for the two lowest temperatures. In the bootstrap loop, one G1/S-ratio point out of the three replicates for each temperature was chosen and used for the calculation. The abundance value was simply the average of the log2(G1/S.ratio) for the first two temperatures (37 and 40.4°C). The stability value was calculated by first subtracting the average log2(G1/S.ratio) of the first temperatures from all log2(G1/S.ratios) of all temperatures and then calculating the sum of the resulting values. After repeating this 500 times, each protein yields a distribution of abundance and stability values. From these distributions we calculated the standard deviation and the average. Together with the number of data points that were considered for bootstrapping, we could calculate a p value (transformation into z-scores and then assuming Student’s t-distribution) to estimate the likelihood that the distribution is different from zero (no change).

The averages were transformed into the z-distribution which represent the new stability and abundance scores. The global FDR was then calculated using the *fdrtool* package.

In order to make a call on significant hits based on these different scores, we chose a cut-off of 0.01 for the local, as well as the global FDR for each cell-cycle and score.

Abundance, expression and stability score data were clustered using proteins that were detected with a significant change in at least one of the different scores in one cell cycle stage. For each protein, each score for each cell cycle stage (18 features) were used as features to calculate the euclidean distance. Using the *Ward.D2* clustering method the results were divided into 21 different clusters which captured most of the variability in the data.

For solubility experiments *limma* calculated p values for comparison between solubility in G1/S (abundance difference between NP40 and SDS) and mitosis (abundance difference between NP40 and SDS) were calculated, and corrected using Benjamin-Hochberg. An FDR cut-off of 0.05 was used, additionally an effect size of > 0.322 (25% regulation) was required for the log2 fold-changes, and the solubility in G1/S had to be < 0.

#### Boxplots

The lower and the upper hinges of the boxes correspond to the 25% and the 75% percentile, and the bar in the box the median. The upper and upper whiskers represent the largest and lowest values, respectively (but at maximum 1.5 times the IQR). Points outside the whiskers are plotted individually. Group tests were performed using a nonparametric Wilcoxon test and statistical significance is indicated by: ^∗^ p ≤ 0.05, ^∗∗^ p ≤ 0.01 and ^∗∗∗^ p ≤ 0.001 if not stated otherwise.

#### Reactome pathway analysis

All quantified proteins from this study were mapped to Reactome pathways (https://reactome.org/download-data/, February 2017) using the *mygene*-package in Python (https://pypi.python.org/pypi/mygene). For each pathway, corrected p values (local FDR) of all its quantified protein components were combined using the Empirical Brown method (EBM) to account for dependent p values (Poole et al., 2016). These combined p values were corrected using the Benjamini-Hochberg procedure. For pathway selection and visualization, we considered those that were sufficiently covered (> 60% pathway coverage with 5-150 quantified protein members), and had at least 30% of its protein components changing significantly in at least one cell cycle stage (local.FDR (stability) < 0.01). 106 pathways that passed these criteria were then manually curated for redundant proteins that changed significantly in several pathways, with 30 pathways retained in the end (visualized in Figure 2D; Table S5). If the combined adjusted p value for a pathway in a given stage was less than 0.05, full opacity is given; otherwise the bubble remains transparent. Overall tendency of a pathway to be stabilized or destabilized was calculated as the median stability score of its significantly changing protein components (local.FDR (stability) < 0.01) in each cell cycle stage.

#### Gene ontology analysis

Gene ontology analysis on proteins significantly affected during the cell cycle in either stability or abundance has been performed with DAVID (version 6.7) (https://david.ncifcrf.gov/), based on GO terms “Biological Process,” “Molecular Function,” “Cellular Compartment” and SP-PIR Keywords. All quantified proteins represented the corresponding background. Benjamini-Hochberg corrected p values were used. For visualization purposes only GO-hits with an FDR < 0.05 were taken into account, and shown in Figure S2C. For Figure 2E only the fold enrichment of the top-hits in each GO-category is shown for each of the 21 clusters.

#### Complex correlation analysis

Proteins were assigned to complexes defined by COMPLEAT and CORUM, as curated and published (Ori et al., 2016). Stability- as well as abundance z-scores were concatenated for each protein, or remained separate vectors, respectively. Subunits of complexes were correlated (Pearson method) with subunits of the same complex; as a control, proteins not assigned to any complex were correlated with other non-complex associated proteins.

#### Complex sub-clustering

For clustering complexes assigned as above stability as well as abundance z-scores were considered. The clustering was performed using the k-medoids algorithm iterating from 2 to 5 potential clusters per complex, and the Euclidean distance between the calculated medoids was monitored at each iteration step. For each complex then the maximum distance reflecting the best possible clustering, is recovered.

#### Analysis of disordered proteins

To assess the nature and origin of the shift in melting points in mitosis relative to the reference, we took into account the disordered state of a protein. The disordered state of each protein was described using the d2p2-database, which combines predictions of several algorithms and makes calls on regions if they have been found to be disordered in at least 75% of those libraries (PONDR VL-XT PONDR VSL2b, PrDOS, PV2, IUPred (+sub-versions of it), Espritz (+sub-versions if it)) (http://d2p2.pro/, February 2017). The protein was ranked according to the relative number of amino acid residues that lie within supposed disordered regions spanning a minimum of 5 amino acids. The relative disordered rank was also corrected taking into account known structural motifs that might overlap with the predicted disordered region using the PDB (see Tables S6 and S7).

#### Mapping of proteins to posttranslational modifications

Modifications were extracted from the PhosphoSitePlus (https://www.phosphosite.org/homeAction.action, March 2017), containing information on known acetylation, methylation, ubiquitination, sumoylation and phosphorylation sites. Proteins containing such sites were scanned for stability and abundance patterns across the cell cycle stages. 512 mitotically regulated phosphorylation sites were extracted from Olsen et al. (2010) (Tables S6 and S7: List of regulated mitotic phosphorylation sites with high occupancy).

#### Organelle/Compartment Analysis

Localization of a protein was defined by the GO-term (only Experimental Evidence is allowed, no inference from computational methods) and the humanProteinAtlas (http://www.proteinatlas.org/, May 2017) using the data supported, validated or approved by antibody-analysis. For the evaluation of the solubility transition data the humanProteinAtlas annotation was used as well as the data from Wilkie et al. (2011) for nuclear envelope annotation.

### Data and Software Availability

All analyzed data is made available in supplemental data files. Supplementary tables and clustering of individual complexes can be found at Mendeley: https://doi.org/10.17632/xrbmvv5srs.2. The MS proteomics data have been deposited to the ProteomeXchange Consortium via the PRIDE partner repository with the dataset identifier PXD008646. All code used for data analysis is available at https://github.com/fstein/cellcycle_TPP.

## Supplemental Information

Table S1. 2D-TPP Cell-Cycle Data, Related to Figure 1

Table S2. Cell-Cycle Scores, Related to Figures 2, 3, 4, and 5

Table S3. SDS Cell-Cycle Data, Related to Figures 2, 4, and 7

Table S4. TPP-TR Data for G1/S and Mitosis, Related to Figures 2, 4, and 6

Table S5. REACTOME Pathways and GO Term Annotation, Related to Figure 2

Table S6. Disordered Proteins, Modifications, Stabilized Set, and Spindle Proteins, Related to Figure 6

Table S7. Protein Solubility, Disorder, and Localization, Related to Figure 7
